# Supplementary material for: Dual ubiquitin signalling by SLOMO controls AUX1 activity and turnover during root gravitropism
Source: EMBO J. 2026 Mar 17;45(8):2739–55. doi: 10.1038/s44318-026-00746-8 (PMC13083858; doi:10.1038/s44318-026-00746-8)
Supplement: Supplementary file 1 — Appendix [file 44318_2026_746_MOESM1_ESM.pdf]

Appendix for

Dual ubiquitin signalling by SLOMO controls AUX1 activity and turnover during root gravitropism

Table of Contents

Appendix Figure S1 – page 3

Appendix Figure S2 – page 4

Appendix Figure S3 – page 5

Appendix Figure S4 – page 6

Appendix Figure S5 – page 7

Appendix Figure S6 – page 8

Appendix Figure S7 – page 9

Appendix Figure S8 – page 10

Appendix Figure S9 – page 11

Appendix Figure S10 – page 12

Appendix Figure S11 – page 13

Appendix Figure S12 – page 14

Appendix Figure S13 – page 15

Appendix Figure S14 – page 15

Appendix Figure S15 – page 17

Appendix Figure S16 – page 18-19

Appendix Figure S17 – page 20

Appendix Figure S18 – page 21

Appendix Figure S19 – page 22

Appendix Figure S20 – page 23

Appendix Figure S21 – page 24

Appendix Figure S22 – page 25

Appendix Figure S23 – page 26

Appendix Figure S24 – page 27-28

Appendix Figure S25 – page 29

Appendix Table S1 – page 30

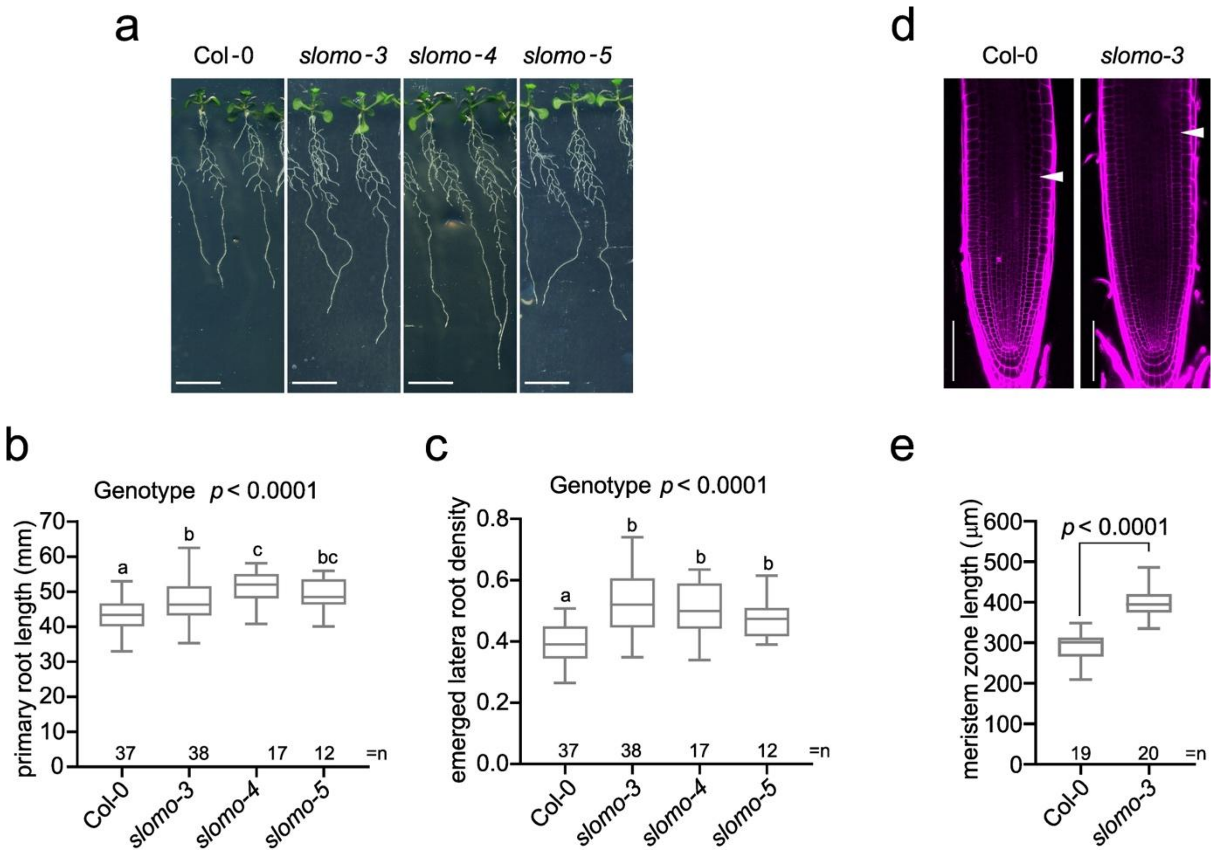

Appendix Figure S1. SLOMO controls primary root growth and root architecture. (**a**) Representative images of Col-0 and *slomo* mutants grown in continuous light conditions for 10 days after germination. Scale bar = 1 cm. (**b-c**) Measurements of primary root length (b) and emerged lateral root density (c). (**d-e**) Representative confocal images (d) and root meristem length quantification (e) of 5 days-after-germination-old *Arabidopsis* seedling roots. Scale bar = 100 µm. The roots were stained with propidium iodide (PI) before imaging. White arrowheads in (d) indicate the end of the meristem zone. Box plots show the median with Tukey-based whiskers and outliers. The number of individually measured seedlings (n) is indicated above the X-axis. Letters indicate significant differences based on one-way ANOVA ($p < 0.05$) (b-c). The *p*-value for the genotype based on one-way ANOVA ($p < 0.05$) (b-c) or on an unpaired t-test with two tailed distributions (e) is shown at the top.

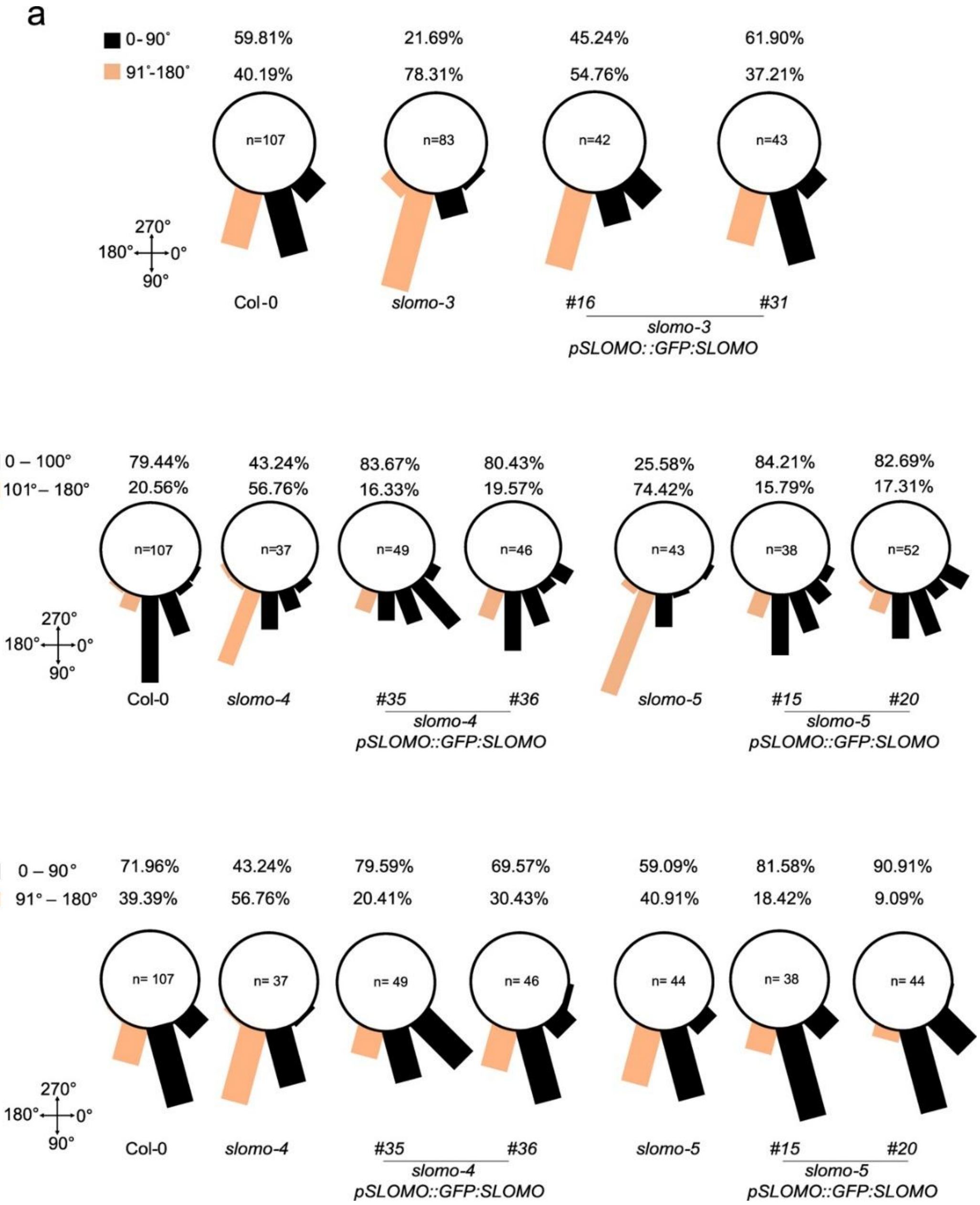

Appendix Figure S2. Distribution of root bending angles after 6 h of gravistimulation of *slomo-3*, *slomo-4*, *slomo-5* and *slomo* lines expressing *pSLOMO::GFP:SLOMO*. Three days-after-germination-old seedlings were rotated 90˚ and were kept in darkness for 6 hours with gravistimulation. The bars surrounding the circle indicate the percentage of root bending angle at 20° intervals (b) and 30° intervals (a, c). The number of individual seedlings examined for each line (n) is shown within the corresponding circles. Root bending angle less than 100° in black, and more than 100° in orange (b). Root bending angle less than 90° in black, and more than 90° in orange (a, c). The 0° starting position upon gravity stimulus and major directions after bending (90°, 180°, and 270°) are indicated. The percentages for each group (as indicated) are shown above the circle.

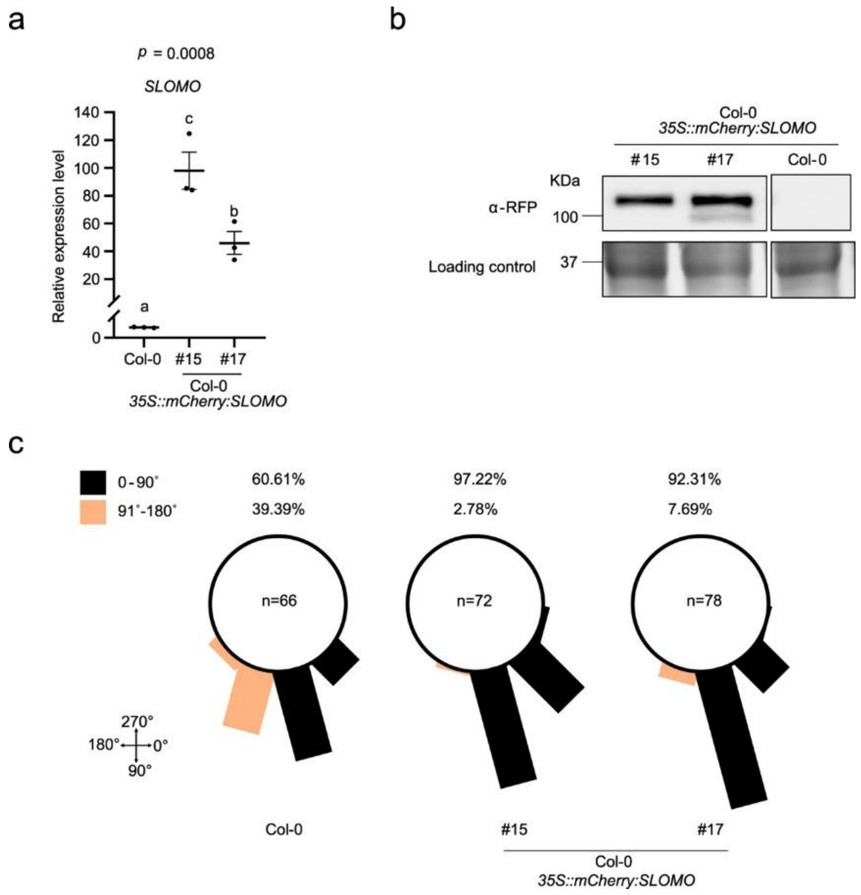

Appendix Figure S3. Characterisation of *35S::mCherry:SLOMO*. (**a**) Relative *SLOMO* expression level in *35S::mCherry:SLOMO*. The diagram shows the mean of 3 biological replicates (individual dots) with a standard error of the mean. Different letters denote significant differences ($p < 0.05$) based on one-way ANOVA with Tukey's HSD. The *p*-value for the genotype is shown at the top. (**b**) Western blot to detect the SLOMO protein level in *35S::mCherry:SLOMO*. mCherry:SLOMO detected by α-RFP antibody. Stain-free gel as loading control. (**c**) Three days-after-germination-old seedlings were rotated 90˚ and were kept in darkness for 6 hours with gravistimulation. The bars surrounding the circle indicate the percentage of root bending angle at 30° intervals. The number of individual seedlings examined for each line (n) is shown within the corresponding circles. Root bending angle less than 90° in black, and more than 90° in orange. The 0° starting position upon gravity stimulus and major directions after bending (90°, 180°, and 270°) are indicated. The percentages for each group (as indicated) are shown above the circle.

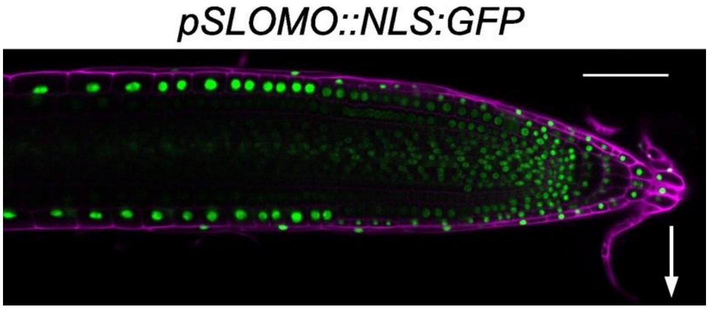

Appendix Figure S4. Expression pattern of *SLOMO* in the primary root tip. Seedlings were stained with propidium iodide (PI) before imaging (magenta signal). The representative image indicates the *SLOMO* expression pattern at the start (0 h) of the gravity stimulus. The arrow indicates the direction of the gravity vector. Scale bar = 75 µm.

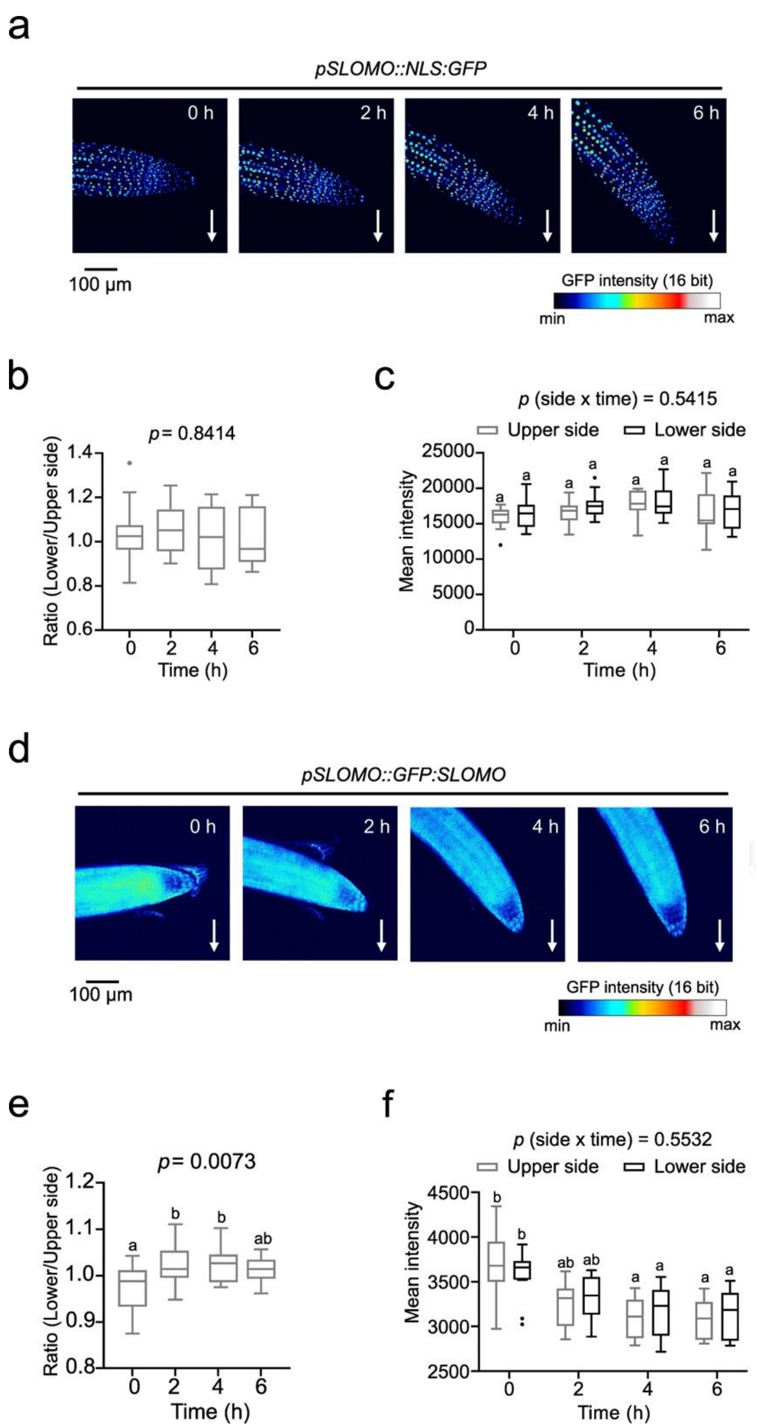

Appendix Figure S5. Live imaging of *SLOMO* expression (a-c) and SLOMO protein distribution (d-f) in the primary root tip upon gravistimulation. Representative image of *pSLOMO:NLS:GFP* (a) and *pSLOMO:GFP:SLOMO* (d) lines, quantification of the fluorescence ratio between the lower and upper root sides during a gravitropic stimulus time series (b,e), and graph of the mean fluorescence intensity at the upper and lower root side (c,f). Box plots show the median with Tukey-based whiskers and outliers. Letters indicate significant differences based on one-way ANOVA ($p < 0.05$). Live imaging and quantification were performed on twelve individual seedlings for each reporter line. Arrows indicate the direction of the direction of the gravity vector. The *p*-values for time (b,e) and for the interaction (side × time) (c,f) are shown at the top.

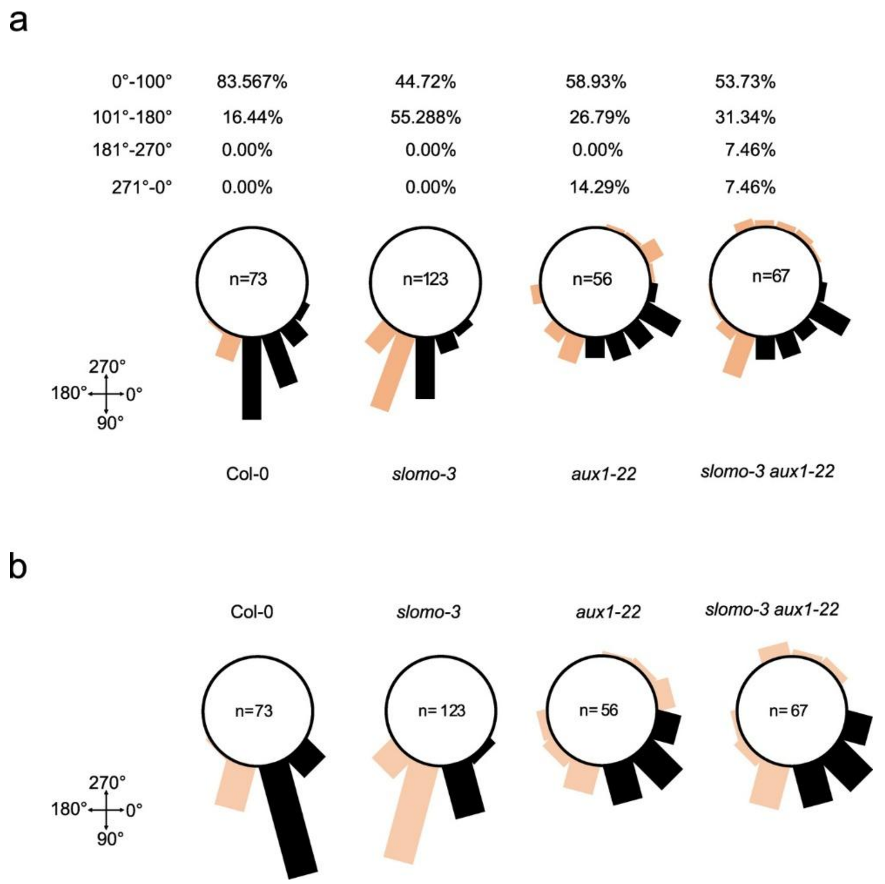

Appendix Figure S6. Distribution of root bending angles after 6 h of gravistimulation of *slomo-3, aux1-22* and *slomo-3 aux1-22*. Three days-after-germination-old seedlings were rotated 90˚ and were kept in darkness for 6 hours with gravistimulation. The bars surrounding the circle indicate the percentage of root bending angle at 20° (a) and 30° (b) intervals. The number of individual seedlings examined for each line (n) is shown within the corresponding circles. Root bending angle less than 100° in black, and more than 100° in orange. The 0° starting position upon gravity stimulus and major directions after bending (90°, 180°, and 270°) are indicated. The percentages for each group (as indicated) are shown above the circle.

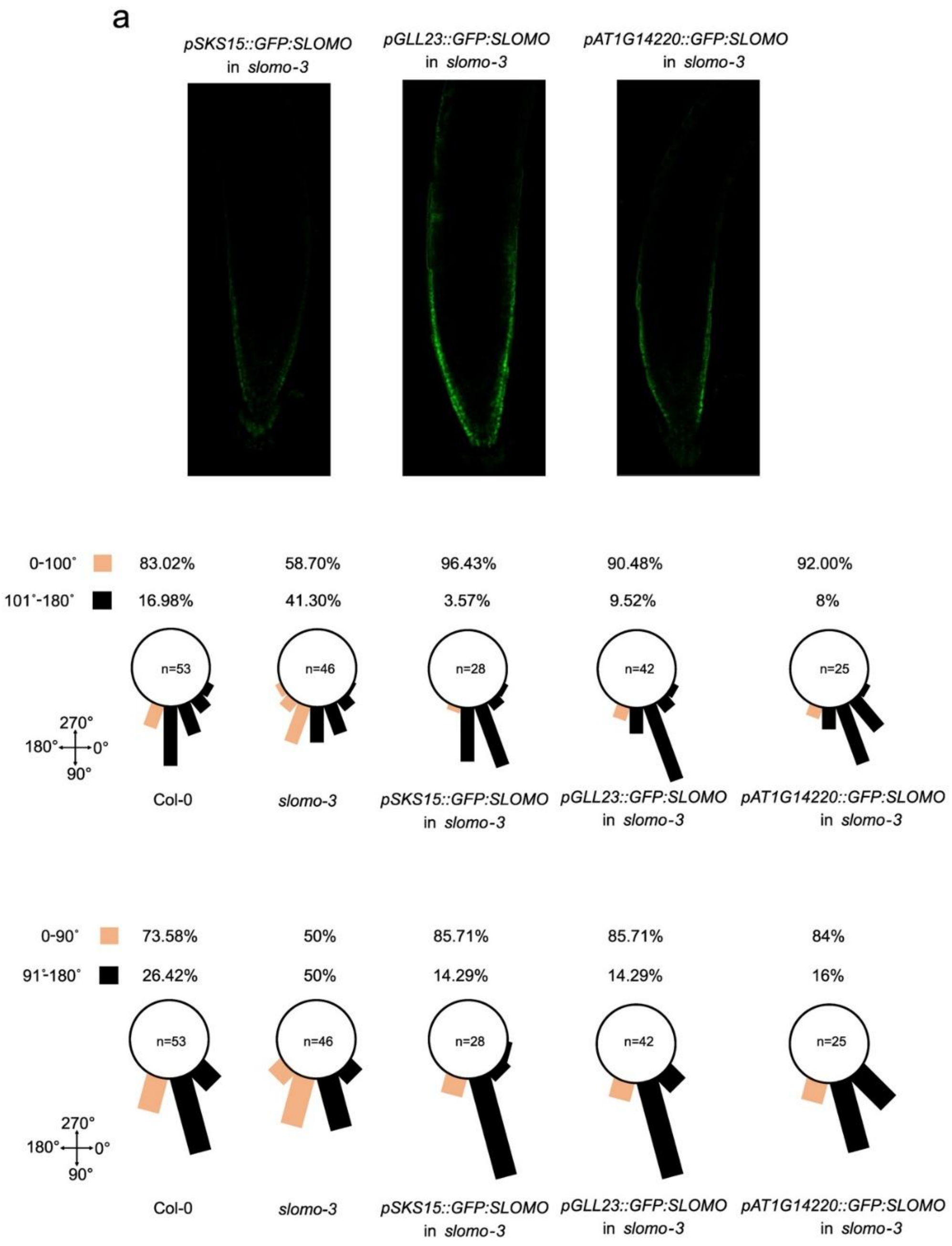

Appendix Figure S7. SLOMO activity in the lateral root cap and/or epidermis is sufficient for its role in root gravitropism. (a) Representative confocal image of expression pattern of *GFP:SLOMO* driven by indicated lateral root cap- and epidermis-specific promotors in primary root of FAST-selected T1 seedlings. **(b-c)** Three days-after-germination-old seedlings were rotated 90° and were kept in darkness for 6 hours with gravistimulation. The bars surrounding the circle indicate the percentage of root bending angle at 20° intervals (b) and 30° intervals (c). The number of individual seedlings examined for each line (n) is shown within the corresponding circles. Root bending angle less than 100° in black, and more than 100° in orange (b). Root bending angle less than 90° in black, and more than 90° in orange (c). The 0° starting position upon gravity stimulus and major directions after bending (90°, 180°, and 270°) are indicated.

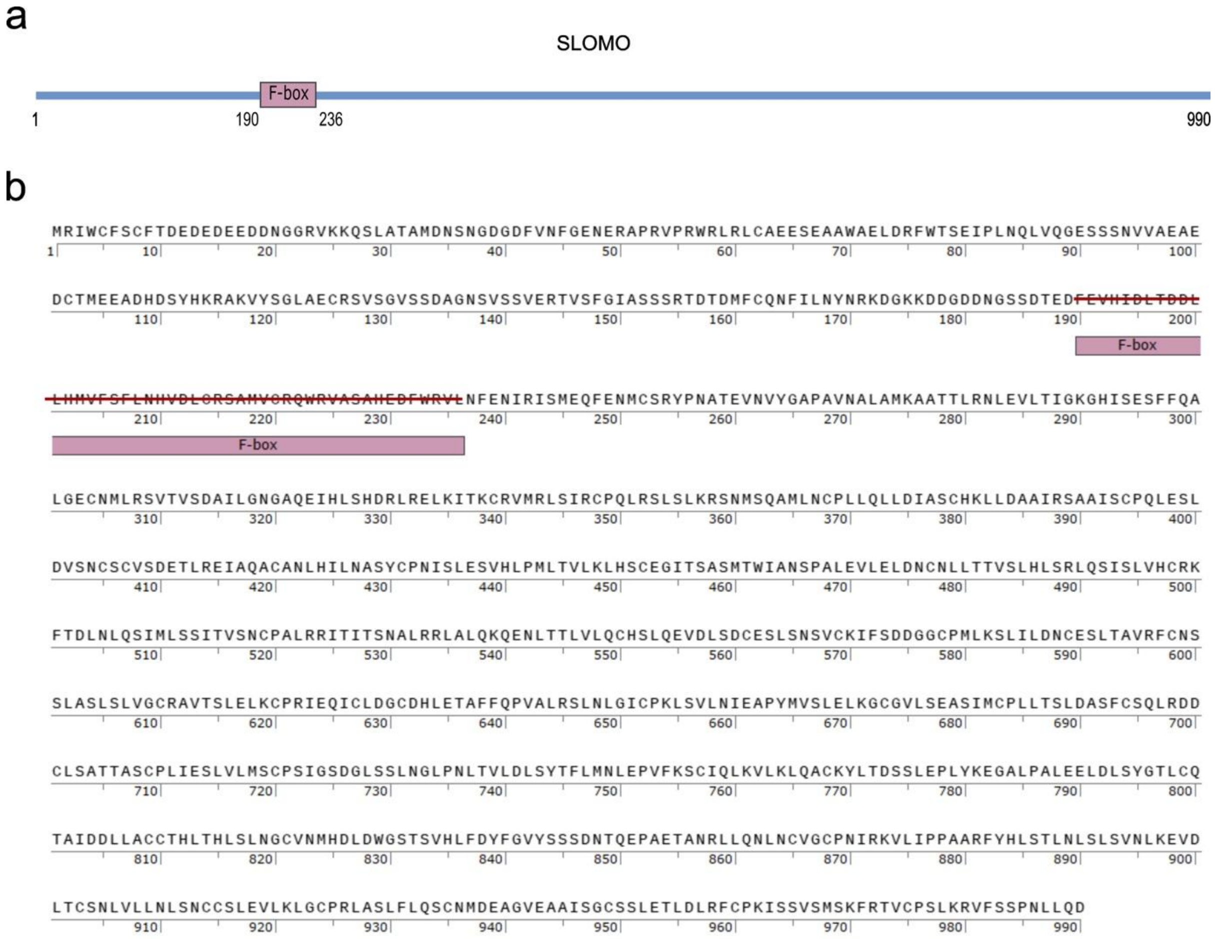

Appendix Figure S8. SLOMO-Decoy protein sequence. (a) Schematic of the SLOMO protein sequence indicating the F-box. **(b)** Detailed protein sequence with the red strike through indicating the F-box that was removed to generate the SLOMO-Decoy protein variant.

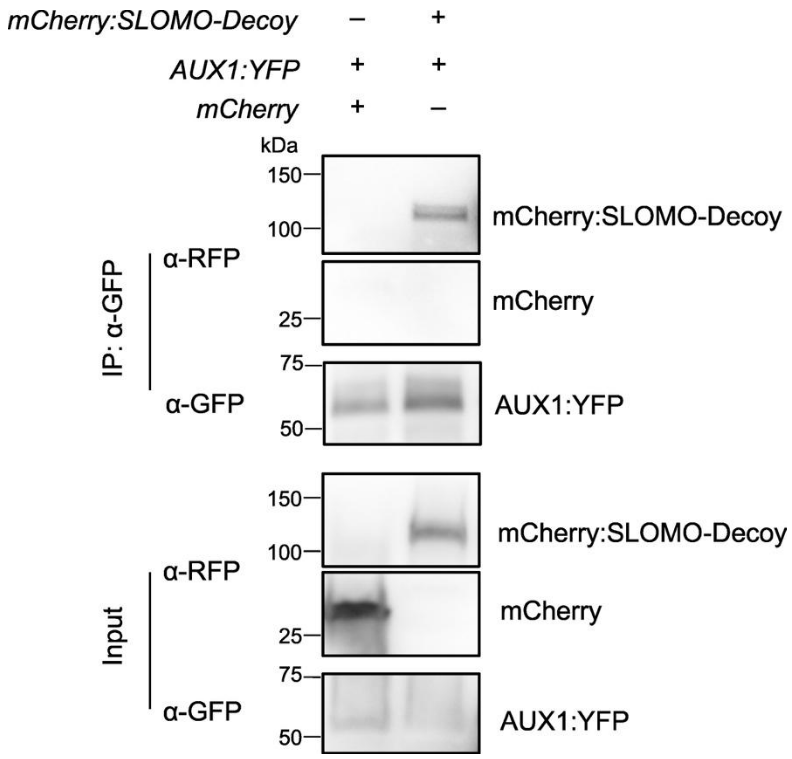

Appendix Figure S9. Western blot of co-IP assay showing that SLOMO does not interact with mCherry upon transient expression in *Nicotiana benthamiana*. All the constructs are driven by the 35S promoter. An α-GFP trap was used for the pull-down and mCherry and mCherry:SLOMO-Decoy were detected using α-RFP.

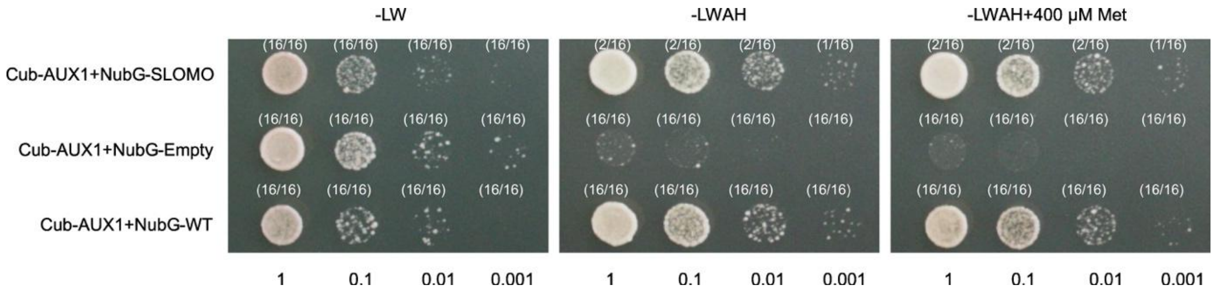

Appendix Figure S10. Split-ubiquitin system in yeast to probe the interaction between AUX1 and SLOMO. AUX1 and SLOMO from *Arabidopsis* were cloned into the bait vector pMETYC and prey vector pNX35-DEST respectively. -LW, -Leu/-Trp; -LWAH, -Ade/-His/-Leu/-Trp; Met, methionine. Soluble NubG-empty (an empty pNX35-DEST) and NubG-WT (an empty pNubWT-Xgate) were used as negative and positive control, respectively. Representative colonies are shown, and the number of similar biological replicates is indicated. Important to note is that we do not see a SLOMO – AUX1 interaction at high frequency (2/16 colonies gave a positive result).

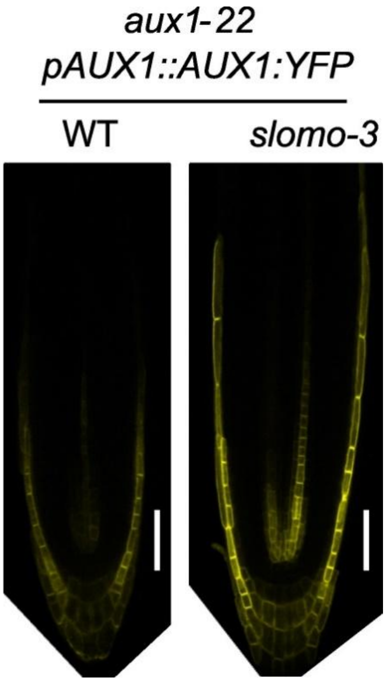

Appendix Figure S11. Representative confocal image of AUX1:YFP signal in wild type and *slomo-3* background. These images are the original and unmodified images used to generate the false colour images in Figure 3C. Scale bar = 50 µm.

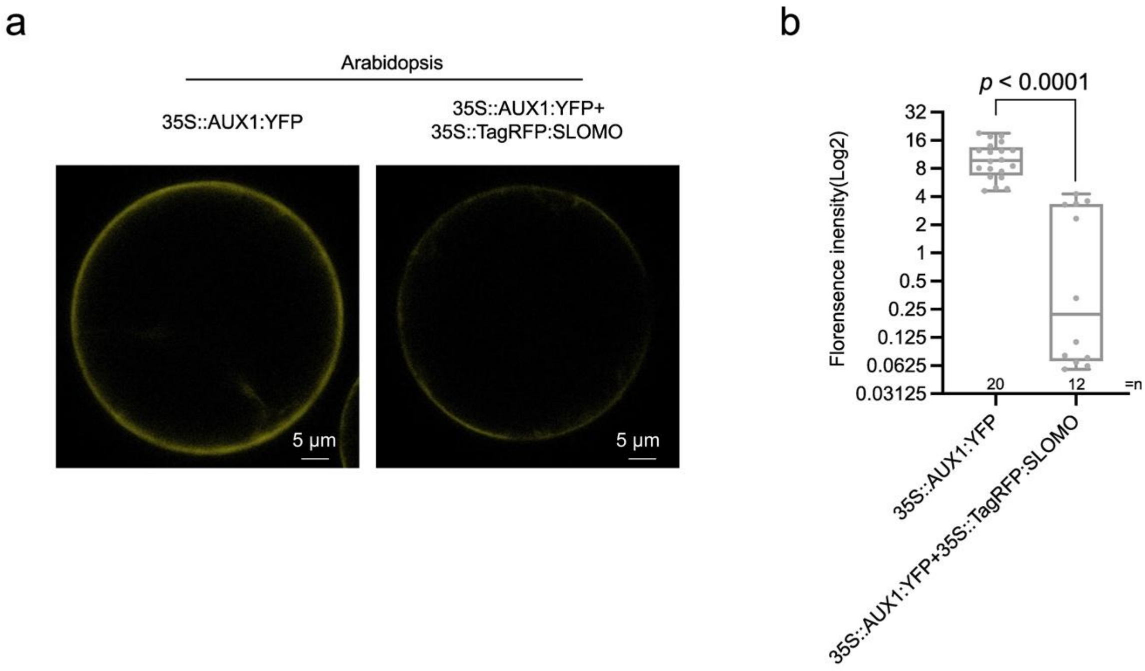

Appendix Figure S12. AUX1 protein levels in the presence of SLOMO upon transient expression in *Arabidopsis* mesophyll protoplasts. (a) Representative mesophyll protoplasts isolated from *Arabidopsis thaliana* Col-0 seedling leaves following transient transformation with either *35S::AUX1:YFP* alone or co-transformation with *35S::AUX1:YFP* and *35S::tagRFP:SLOMO*. Scale bar = 5 µm. (**b)** Quantification of AUX1:YFP fluorescence. Box plots show whiskers down to the minimum and up to the maximum value and each individual value is shown as a dot superimposed on the graph. Y-axis values are presented on a log2 scale. The number of individually measured protoplasts (n) is indicated above the X-axis. The *p*-value based on unpaired t-test with two-tailed distribution is shown at the top.

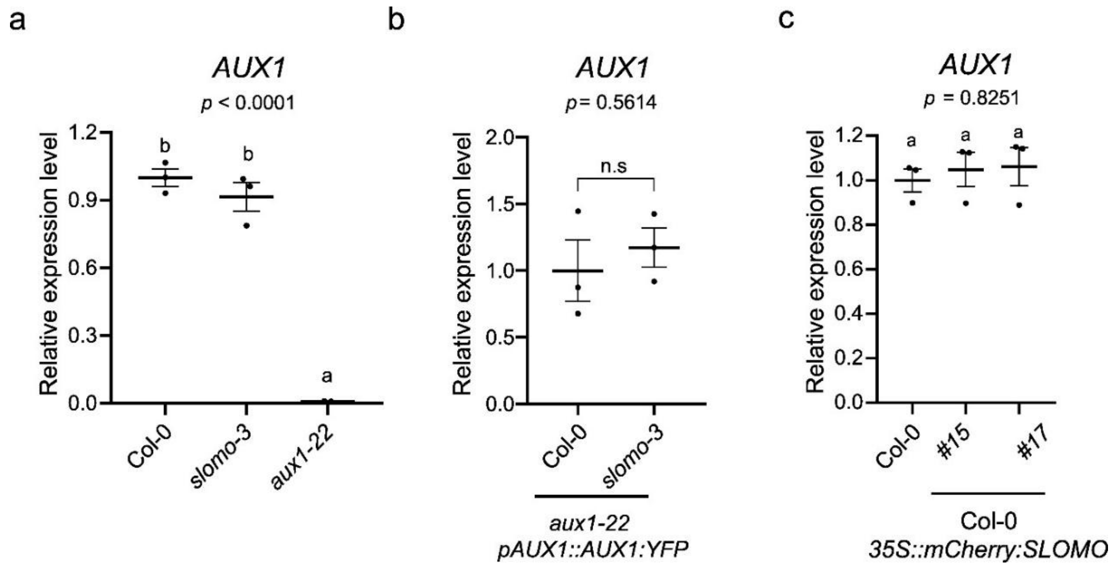

Appendix Figure S13. ***AUX1*** **expression level in** ***slomo-3*** **and** ***35S::mCherry:SLOMO*****.** The graphs show the mean of three biological replicates (individual dots) with standard error. Different letters denote significant differences ($p < 0.05$) based on one-way ANOVA with Tukey's HSD (a, c), while same letters indicate no significant differences ($p > 0.05$). "n.s." indicates no significant difference based on an unpaired t-test ($p > 0.05$) (b). The *p*-values for each genotype are shown at the top.

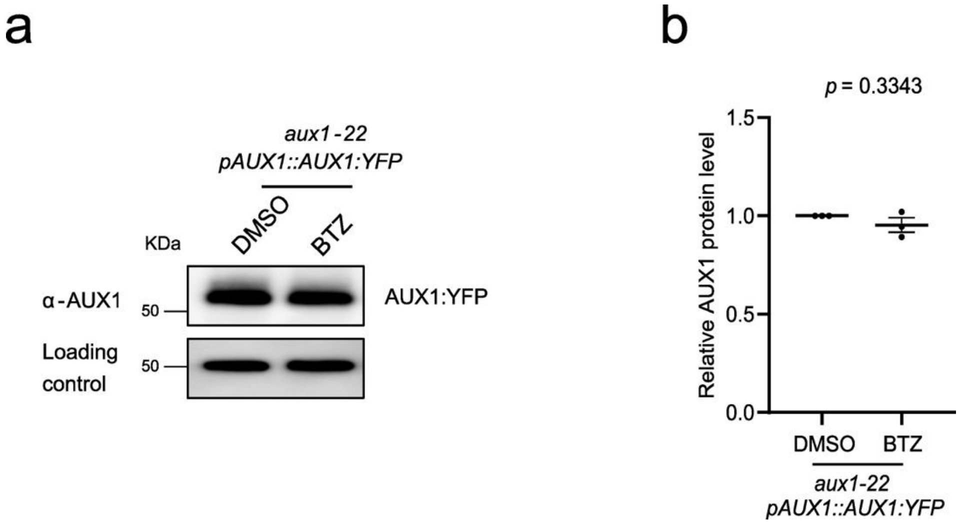

Appendix Figure S14. Effect of bortezomib (BTZ) treatment on AUX1:YFP levels. (a) Representative Western blot showing AUX1 abundance after 40 μM BTZ treatment (a). Loading control, bands detected by α-Tubulin. **(b)** Quantification of the relative level of AUX1 (AUX1/loading control). The graph shows the mean of 3 biological replicates (individual dots) with standard error of the mean. No significant differences ($p < 0.05$) based on a paired t-test with two-tailed distribution. The *p*-value is shown above the graph. The experiment was repeated three times with similar results.

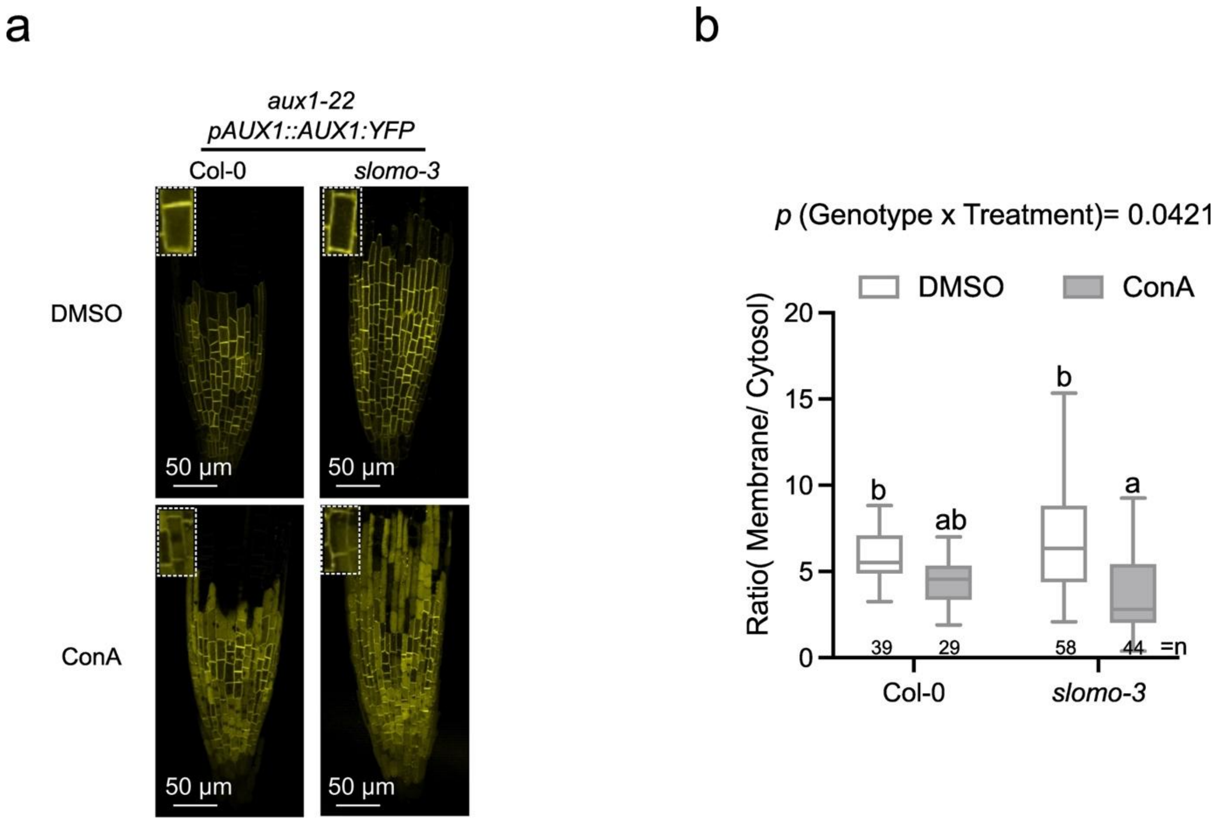

Appendix Figure S15. AUX1:YFP localisation in wild-type Col-0 and *slomo-3*. (**a**) Representative confocal images of root cells from Col-0 and *slomo-3* seedlings expressing *pAUX1::AUX1:YFP*. Inset highlights one individual cell. Five-day-after-germination-old seedlings were incubated in liquid MS medium with DMSO or 1 µM ConA under dark conditions for 16 hours. Scale bar = 50 µm. (**b)** AUX1:YFP signal ratios between plasma membrane and cytosol in DMSO and ConA-treated seedlings for individual cells. Box plots show the median with Tukey-based whiskers. Different letters denote significant differences (p < 0.05) based on two-way ANOVA with Tukey's HSD. The number of individually measured cells (n) is indicated above the X-axis. The *p*-value for Genotype x Treatment is indicated above the graph.

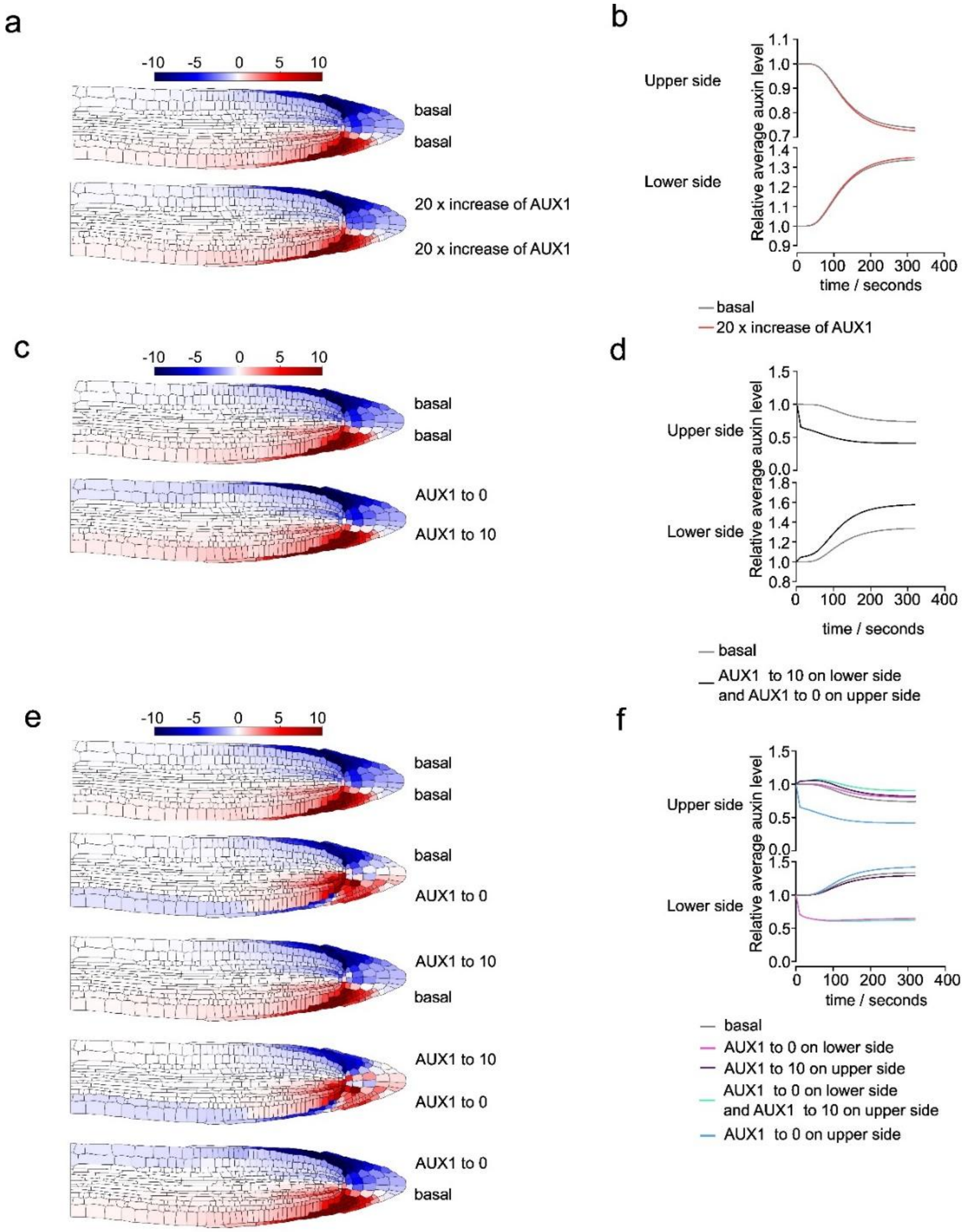

Appendix Figure S16. Model predictions for auxin (re)distribution in the root tip upon gravitropic stimulus. (a-b) Auxin redistribution in the root tip with basal AUX1 levels and with 20 times overall increase in AUX1 levels. **(c-d)** Auxin redistribution in the root tip with upper side decreased to 0 and lower side increased to 10 (maximum of AUX1 level). **(e-f)** Auxin redistribution in the root tip with lower side decreased to 0, with upper side increased to 10, with upper side increased to 10 and lower side decreased to 0, and with upper side decreased to 0. The root tip drawings give the auxin distribution at time point 5 min in the mathematical model (a, c, and e). The graphs capture the change in auxin levels in the upper versus the lower part of the root tip over time (b, d and f). The multicellular root templates show the auxin

distribution at time point 5 min predicted by the mathematical model. The graphs show the predicted change in epidermal auxin levels in the upper versus the lower part of the root tip over time, calculated as the average auxin concentration in the transition-zone epidermal cells. The relative auxin level is the ratio of the average auxin concentration in the transition-zone epidermal cells to the auxin concentration at the initial time point. It should be noted that the image showing AUX1 redistribution in root tips with basal AUX1 levels is the same control reused for all comparisons in a, c, and e, and also in Figure 3I.

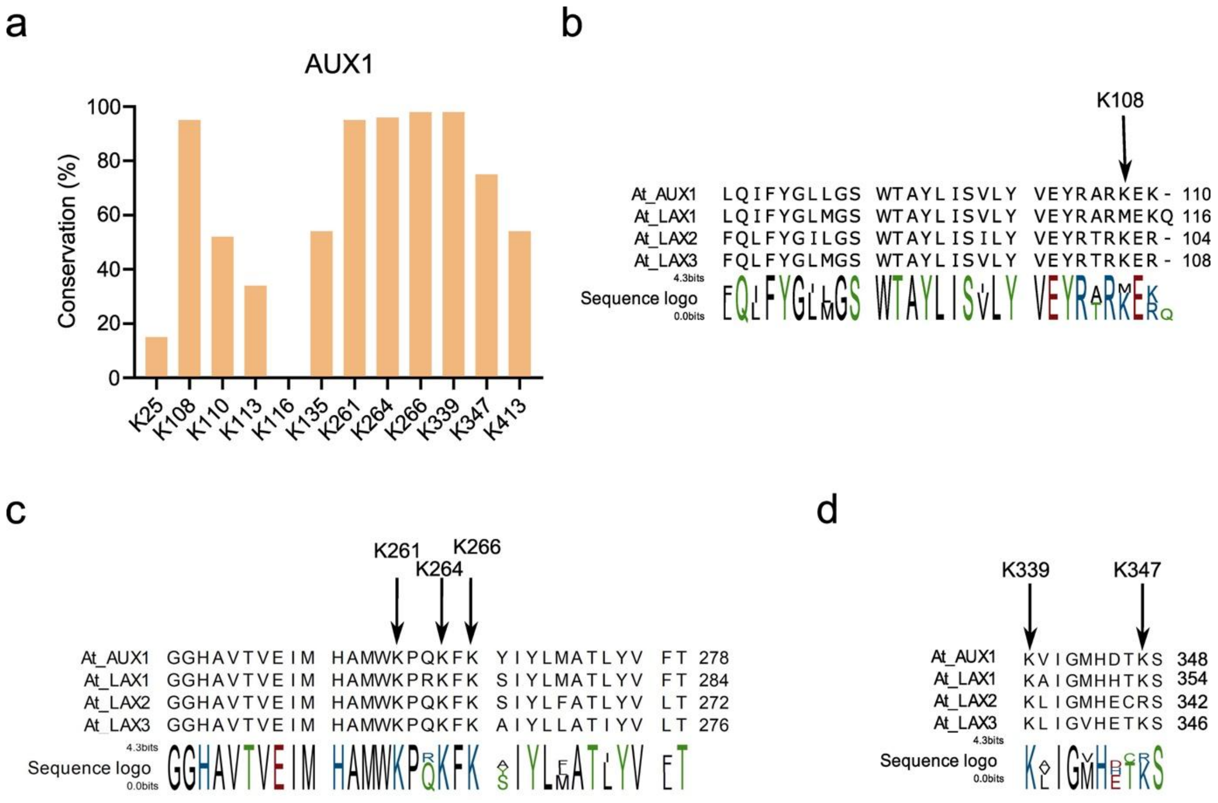

Appendix Figure S17. Conserved AUX1 Lysines in the cytosol. (**a**) The bar diagram shows the conservation of detected lysines among 570 orthologous genes with 96 species. (**b-d**) Alignment of AUX1 fragment containing AUX1^{K108} (b) or AUX1^{K261}, AUX1^{K264} and AUX1^{K266} (c) or AUX1^{K339} and AUX1^{K347} (d) among Arabidopsis AUX1/LAX family members.

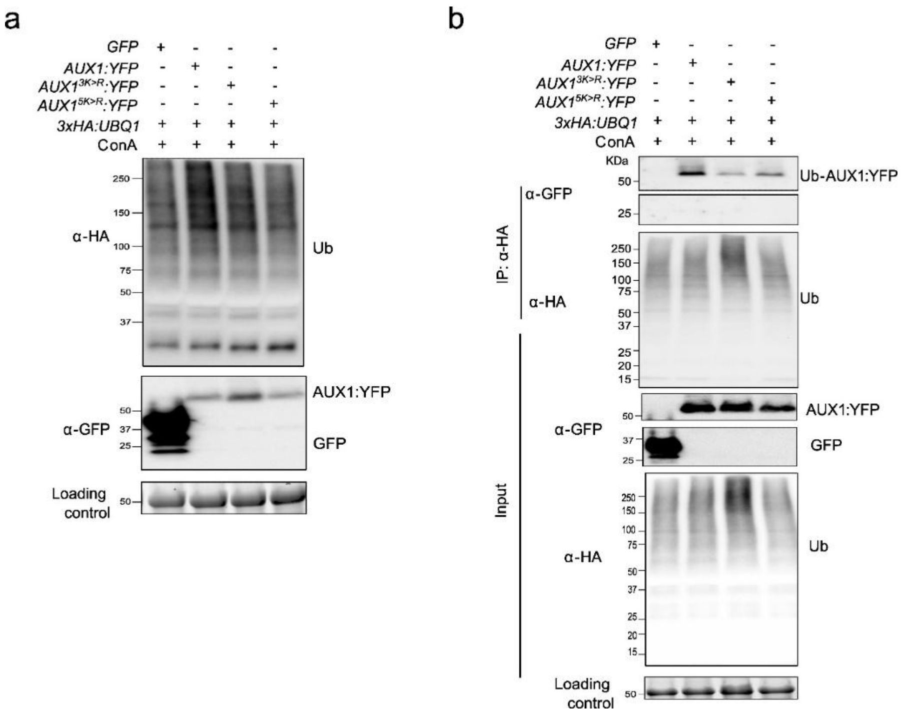

Appendix Figure S18. Ubiquitination of AUX1 and AUX1 variants with K>R exchanges. **(a)** Input western blot pictures of Fig. 4b. (**b**) Representative Western blot detecting Ub-AUX1 following YFP:AUX1 immunoprecipitation (IP) upon transient expression of *AUX1*, $AUX1^{3K>R}$ or $AUX1^{5K>R}$ with *HA:UBQ1* in *N. benthamiana*. IP products were precipitated with an anti-HA antibody. IP products were probed with an anti-HA or anti-GFP antibody. Experiments were performed in the presence of 1 µM ConA. The experiment was repeated at least two times with similar results.

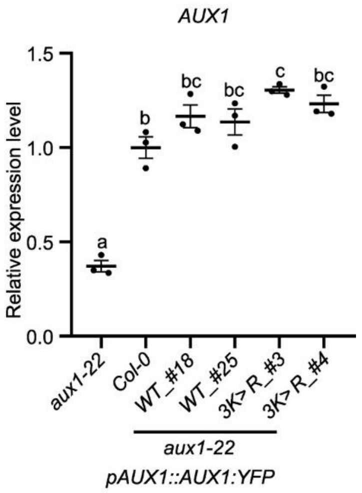

Appendix Figure S19. ***AUX1*** **expression level in** ***aux1*** **lines expressing AUX1 variants with 3K>R exchanges.** The graph shows the mean of 3 biological replicates (individual dots) with a standard error of the mean. Different letters denote significant differences ($p < 0.05$) based on one-way ANOVA with Tukey's HSD.

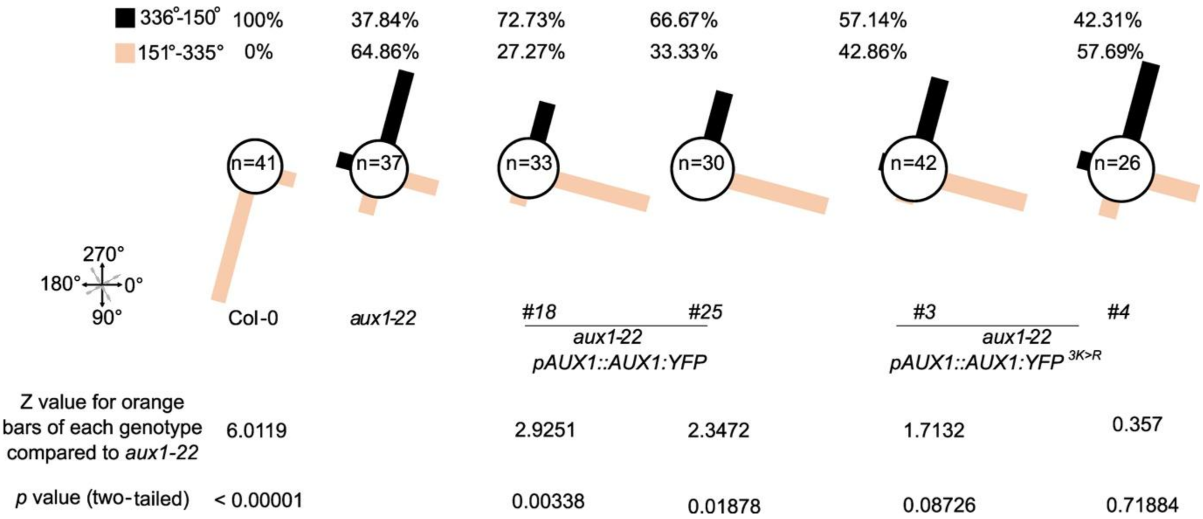

Appendix Figure S20. Distribution of root bending angles after 6 h of gravistimulation of *aux1-22* expressing *pAUX1::AUX1:YFP* or *pAUX1::AUX1*$^{3K>R}$*:YFP*. Three days-after-germination-old seedlings were rotated 90˚ and were kept in darkness for 6 hours with gravistimulation. The bars surrounding the circle indicate the percentage of root bending angle at 60° intervals (according to the grey dashed line indicated on the cross). The number of individual seedlings examined for each line (n) is shown within the corresponding circles. Root bending angles for 336°-150° in black, and for 151°-335° in orange. The percentages for each group (as indicated) are shown above the circle. The 0° starting position upon gravity stimulus and major directions after bending (90°, 180°, and 270°) are indicated. A Z-test for two population proportions was used to determine whether the 151°–335° group of each line differs significantly from *aux1-22*. The Z value and associated *p*-value are indicated.

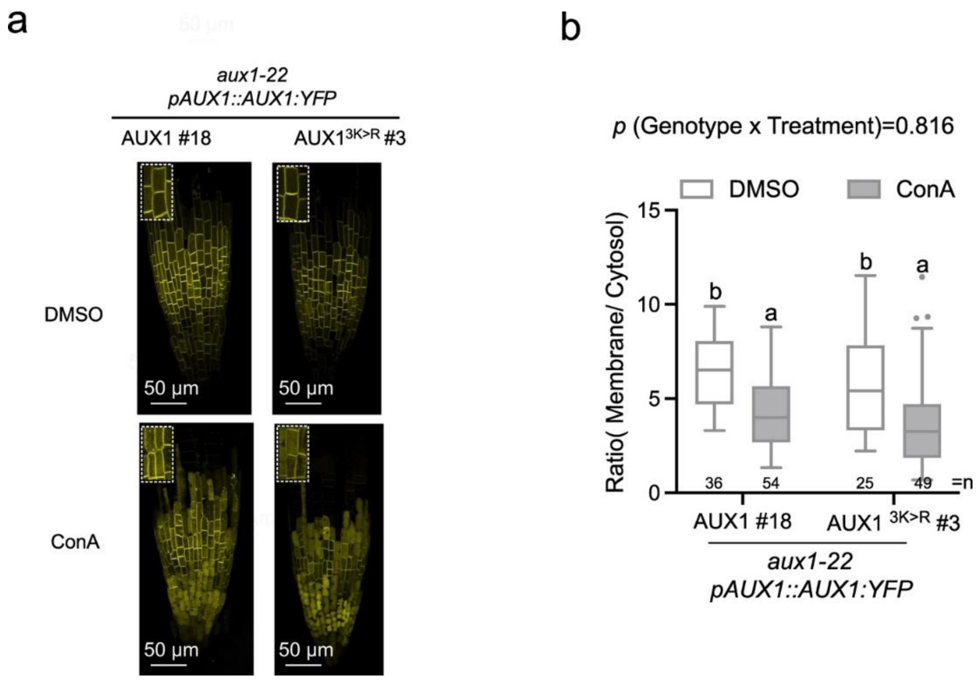

Appendix Figure S21. AUX1:YFP localisation in wild-type Col-0 and *slomo-3*. (**a**) Representative confocal images of root cells from *aux1-22* seedlings expressing *pAUX1::AUX1:YFP* or *pAUX1::AUX1*$^{3K>R}$*:YFP*. Inset highlights one individual cell. The 5-day-after-germination-old seedlings were incubated in liquid MS medium with DMSO or 1 µM ConA under dark conditions for 16 hours. Scale bar = 50 µm. (**b**) AUX1:YFP signal ratios between plasma membrane and cytosol in DMSO and ConA-treated seedlings for individual cells. Box plots show the median with Tukey-based whiskers. Different letters denote significant differences ($p < 0.05$) based on two-way ANOVA with Tukey's HSD. The number of individually measured cells (n) is indicated above the X-axis. The *p*-value for Genotype x Treatment is indicated above the graph.

a

b

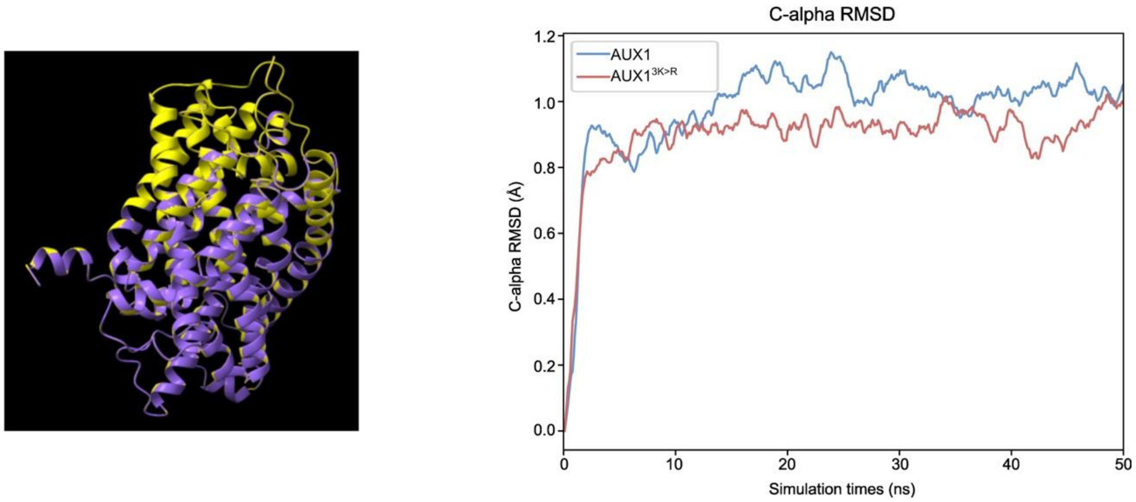

Appendix Figure S22. The structural motion of AUX1 and AUX1$^{3K>R}$ using molecular dynamics simulation. (**a**) Protein structure alignment between AUX1 (yellow) and AUX1$^{3K>R}$ (purple). (**b**) Root-mean-square deviation (RMSD) of alpha carbon (Cα) of the overall AUX1 and AUX1$^{3K>R}$ structures within a 50-ns simulation.

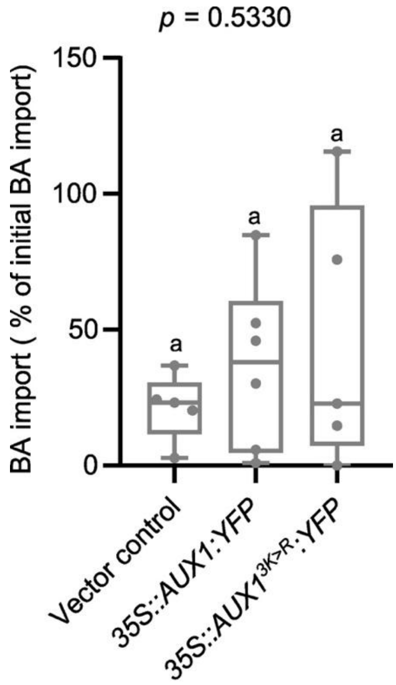

Appendix Figure S23. Import assay for 14C-benzoic acid (BA) in protoplasts prepared from tobacco leaves transfected with *35S::AUX1:YFP* and *35S::AUX1$^{3K>R}$:YFP*. The box plots with individual data points represent the distribution of all individual samples (n=5), showing the median (central lines) with Tukey-based whiskers. Letters indicate significant differences based on one-way ANOVA (p<0.05). The *p*-value is shown at the top.

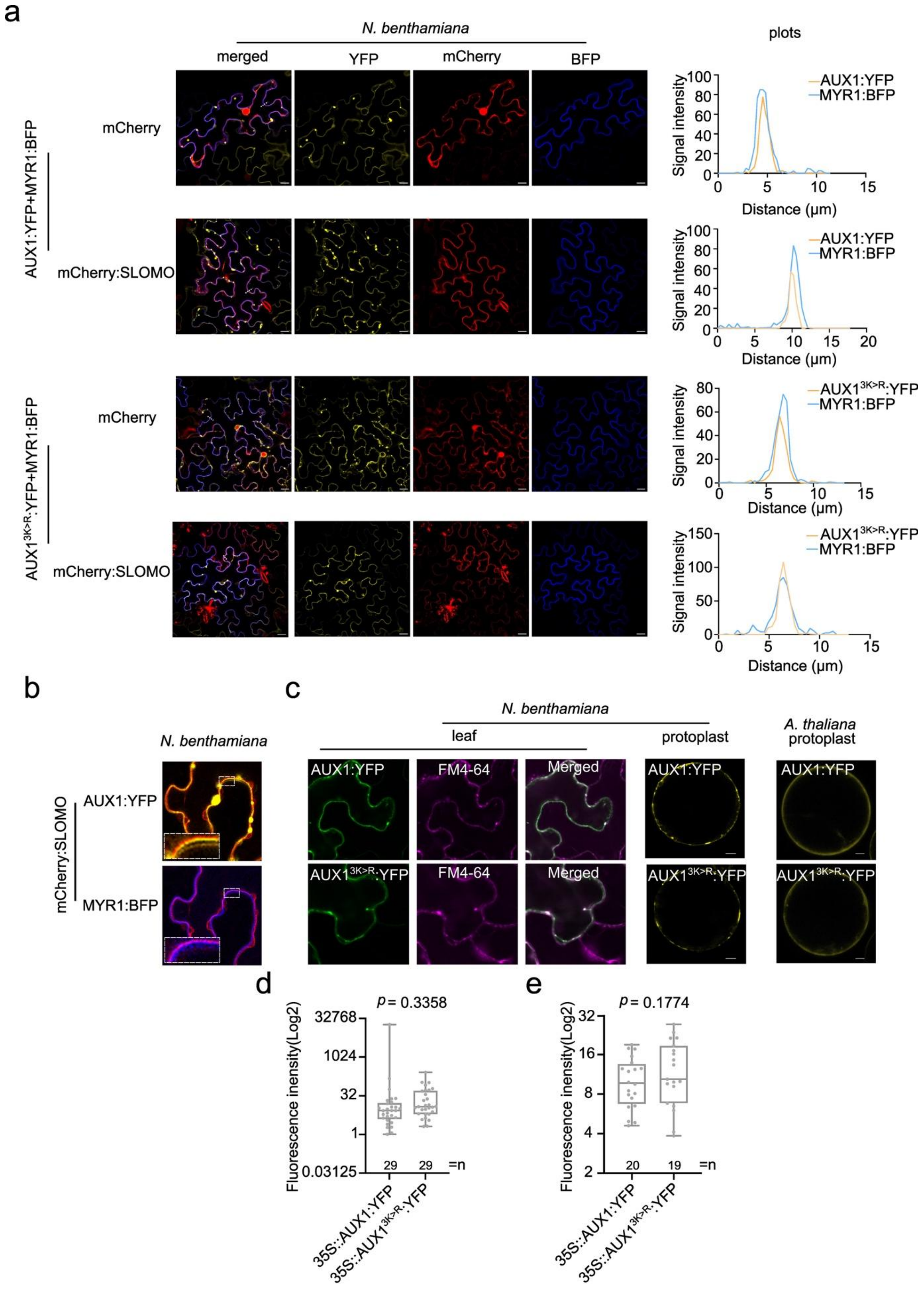

Appendix Figure S24. Subcellular localization of AUX1:YFP and AUX1$^{3K>R}$:YFP. (a) Representative images of co-localization of AUX1:YFP and AUX1$^{3K>R}$:YFP with the plasma membrane marker MYR1:BFP, in the presence of mCherry or mCherry:SLOMO, when

transiently co-expressed in *N. benthamiana* leaves. The fluorescence intensity profiles along the white dashed lines are shown on the right. Scale bar = 20 μm. (**b)** Representative images of co-localization of mCherry:SLOMO (red) and AUX1:YFP (yellow) or plasma membrane marker MYR1:BFP (blue) in *Nicotiana benthamiana* leaves. Magnifications of the boxed regions are shown as inset. **(c)** Subcellular localization of AUX1:YFP and AUX1$^{3K>R}$:YFP in *Nicotiana benthamiana* leaves. Leaves were stained with the plasma membrane marker FM4-64 prior to imaging. Localization in mesophyll protoplasts derived from *N. benthamiana* and *A. thaliana*. Scale bar = 10 μm (*N. benthamiana* protoplast) or 5 μm (*A. thaliana* protoplast). **(d-e)** Quantification of fluorescence from AUX1:YFP or AUX1$^{3K>R}$:YFP for protoplasts from *N. benthamiana* (d) and *Arabidopsis thaliana* (e) respectively. The box plots with individual data points represent the distribution of all individual protoplasts, showing the median (central lines) with Tukey-based whiskers. Y-axis values are presented on a $\log_2$ scale. The *p*-values from unpaired t-tests with two tailed distribution are shown at the top. The number of individually measured protoplasts (n) is indicated above the X-axis.

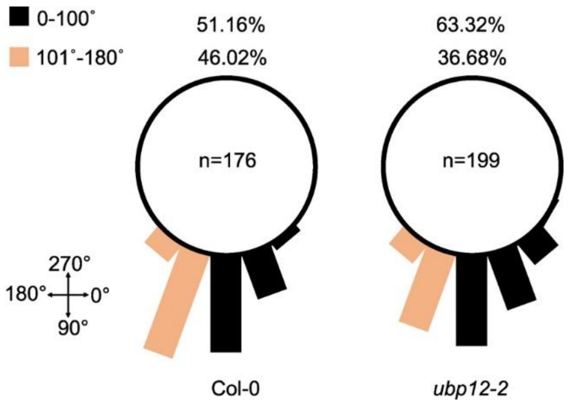

Appendix Figure S25. Distribution of root bending angles after 6 h of gravistimulation of *ubp12-2 mutant.* Three days-after-germination-old seedlings were rotated 90˚ and were kept in darkness for 6 hours with gravistimulation. The bars surrounding the circle indicate the percentage of root bending angle at 20° intervals. The number of individual seedlings examined for each line (n) is shown within the corresponding circles. Root bending angle less than 100° in black, and more than 100° in orange. The 0° starting position upon gravity stimulus and major directions after bending (90°, 180°, and 270°) are indicated. The percentages for each group (as indicated) are shown above the circle.

**Appendix Table S1. Primers used in this study**			
**Primer ID**	**Sequence**	**Application**	**Note**
ARP7-Fwd	ACTCTTCCTGATGGACAGGTG	RT-qPCR	Housekeeping gene
ARP7-Rev	CTCAACGATTCCATGCTCCT	RT-qPCR	Housekeeping gene
EF1α-Fwd	CTGGAGGTTTTGAGGCTGGTAT	RT-qPCR	Housekeeping gene
EF1α -Rev	CCAAGGGTGAAAGCAAGAAGA	RT-qPCR	Housekeeping gene
SLOMO-Fwd	ATGGAGCGCAGGAAATACAC	RT-qPCR	
SLOMO-Rev	GGATAGCAGCATCCAAGAGC	RT-qPCR	
AUX1-Fwd	GAGGTCACGCGGTTACTGTT	RT-qPCR	
AUX1-Rev	GAGAGAAAGCGTTGGAGTGG	RT-qPCR	
AUX1-Fwd	ATGTCGGAAGGAGTAGAAGC	PCR	
AUX1-Rev	TCAAAGACGGTGGTGTAAAGCGG	PCR	
AUX1_attB1	GGGGACAAGTTTGTACAAAAAAGCAGGCTCCATGTCGGAAGGAGTAGAAGC	Gateway cloning	
AUX1_ stop_attB2	GGGGACCACTTTGTACAAGAAAGCTGGGTCAAGACGGTGGTGTAAAGCGG	Gateway cloning	
LAX1_attB1	GGGGACAAGTTTGTACAAAAAAGCAGGCTCCATGTCGGGTGAGAAACAAGC	Gateway cloning	
LAX1_Stop_attB2	GGGGACCACTTTGTACAAGAAAGCTGGGTCCTAACGGCGGTGGTGAGCTC	Gateway cloning	
LAX2_attB1	GGGGACAAGTTTGTACAAAAAAGCAGGCTCCATGGAGAACGGTGAGAAAGC	Gateway cloning	
LAX2_stop_attb2	GGGGACCACTTTGTACAAGAAAGCTGGGTCTCAAAGGCCGTGAGTGTGAT	Gateway cloning	
