# Supplementary figures and images for: Dual ubiquitin signalling by SLOMO controls AUX1 activity and turnover during root gravitropism
Source: EMBO J. 2026 Mar 17;45(8):2739–55. doi: 10.1038/s44318-026-00746-8 (PMC13083858; doi:10.1038/s44318-026-00746-8)

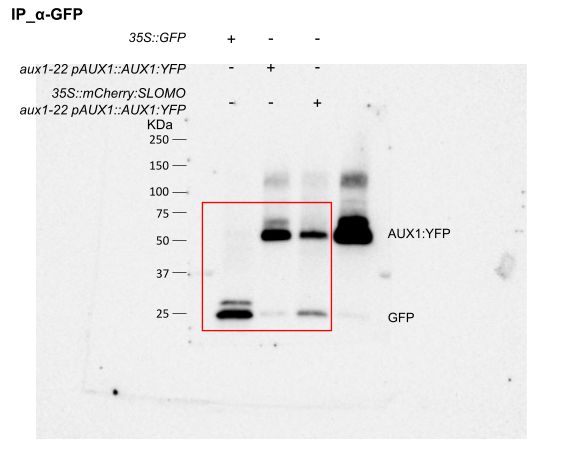

Supplement: Supplementary file 4 — Source data Fig. 2 [file 44318_2026_746_MOESM4_ESM.zip › Figure 2 Source Data/Figure 2B/Rep1/IP_Anti-GFP.tiff]

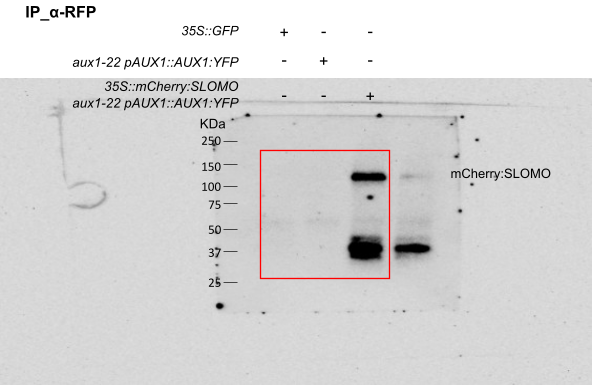

Supplement: Supplementary file 4 — Source data Fig. 2 [file 44318_2026_746_MOESM4_ESM.zip › Figure 2 Source Data/Figure 2B/Rep1/IP_Anti-RFP.tiff]

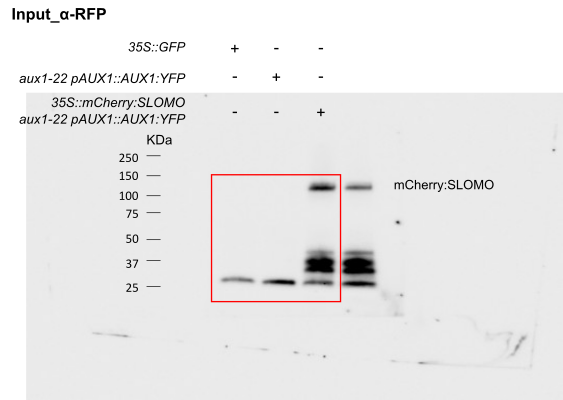

Supplement: Supplementary file 4 — Source data Fig. 2 [file 44318_2026_746_MOESM4_ESM.zip › Figure 2 Source Data/Figure 2B/Rep1/Input_Anti-RFP.tiff]

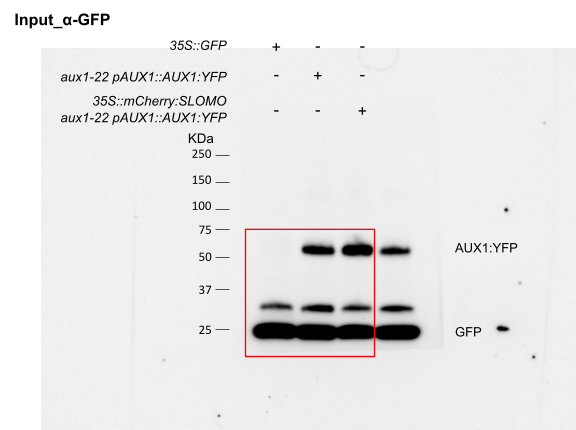

Supplement: Supplementary file 4 — Source data Fig. 2 [file 44318_2026_746_MOESM4_ESM.zip › Figure 2 Source Data/Figure 2B/Rep1/Input_Anti-GFP.tiff]

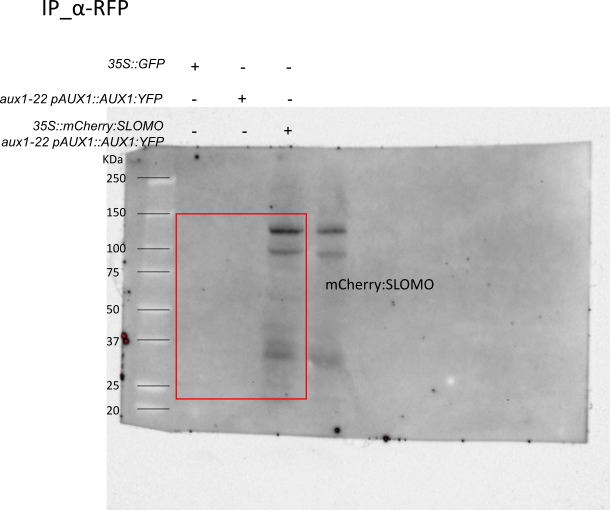

Supplement: Supplementary file 4 — Source data Fig. 2 [file 44318_2026_746_MOESM4_ESM.zip › Figure 2 Source Data/Figure 2B/Rep2/IP_anti_RFP.tiff]

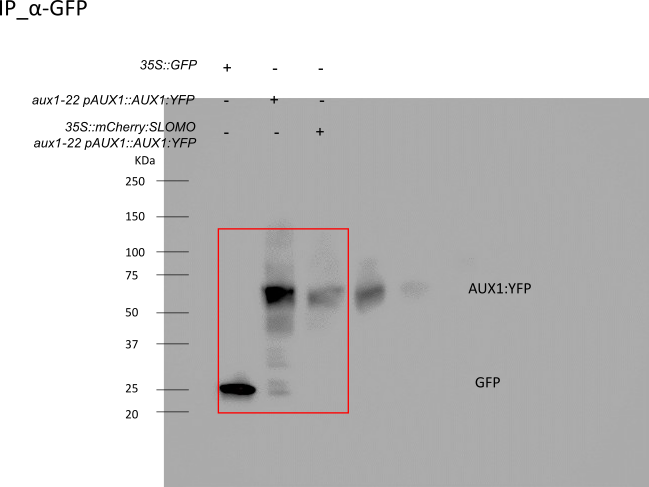

Supplement: Supplementary file 4 — Source data Fig. 2 [file 44318_2026_746_MOESM4_ESM.zip › Figure 2 Source Data/Figure 2B/Rep2/IP_Anti_GFP.tiff]

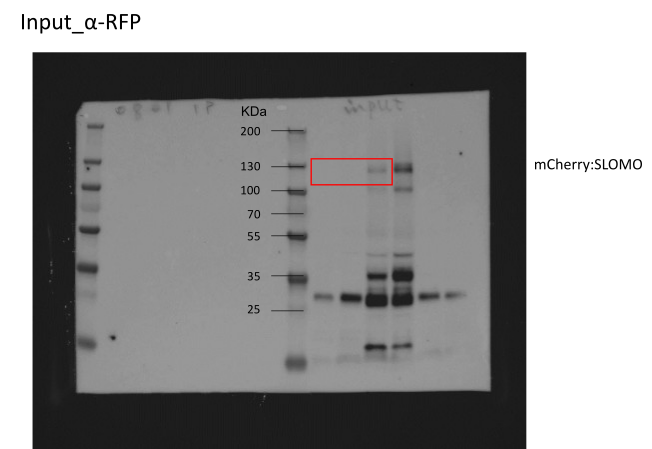

Supplement: Supplementary file 4 — Source data Fig. 2 [file 44318_2026_746_MOESM4_ESM.zip › Figure 2 Source Data/Figure 2B/Rep2/Input_RFP.tiff]

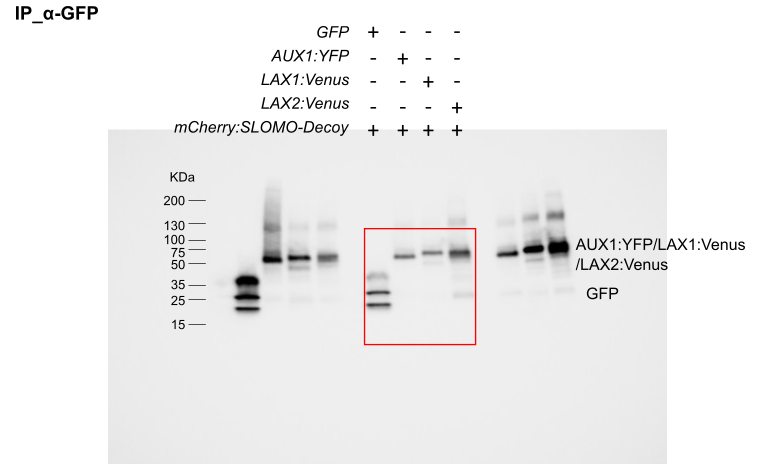

Supplement: Supplementary file 4 — Source data Fig. 2 [file 44318_2026_746_MOESM4_ESM.zip › Figure 2 Source Data/Figure 2A/IP_Anti-GFP.tiff]

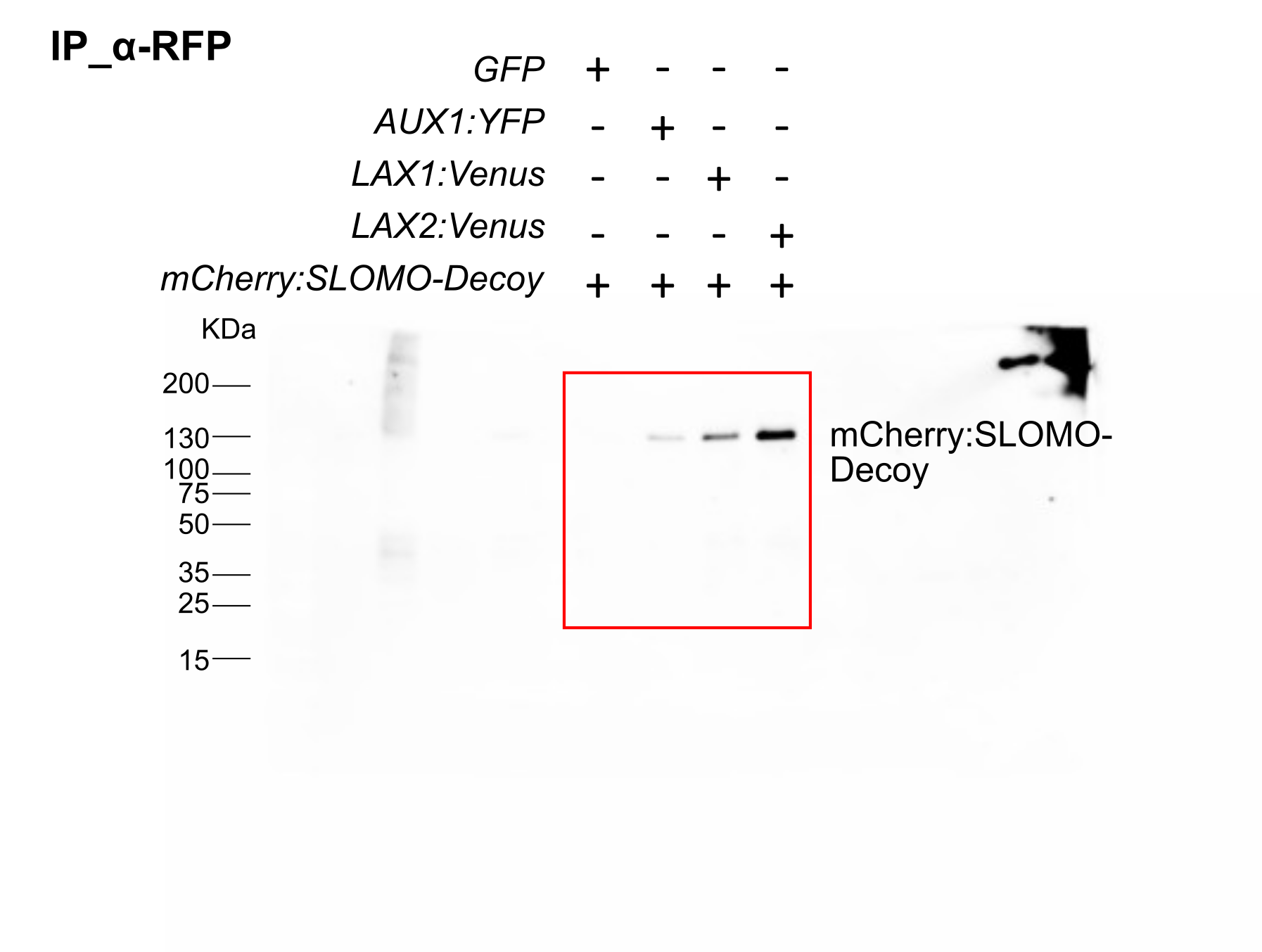

Supplement: Supplementary file 4 — Source data Fig. 2 [file 44318_2026_746_MOESM4_ESM.zip › Figure 2 Source Data/Figure 2A/IP_Anti-RFP.tiff]

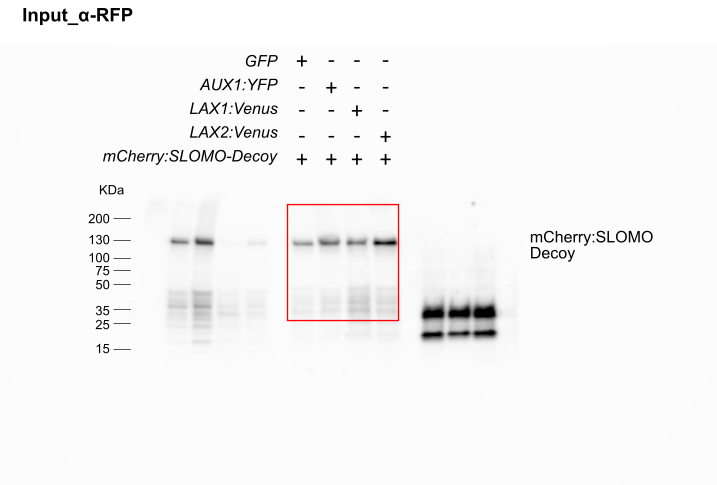

Supplement: Supplementary file 4 — Source data Fig. 2 [file 44318_2026_746_MOESM4_ESM.zip › Figure 2 Source Data/Figure 2A/Input_Anti-RFP.tiff]

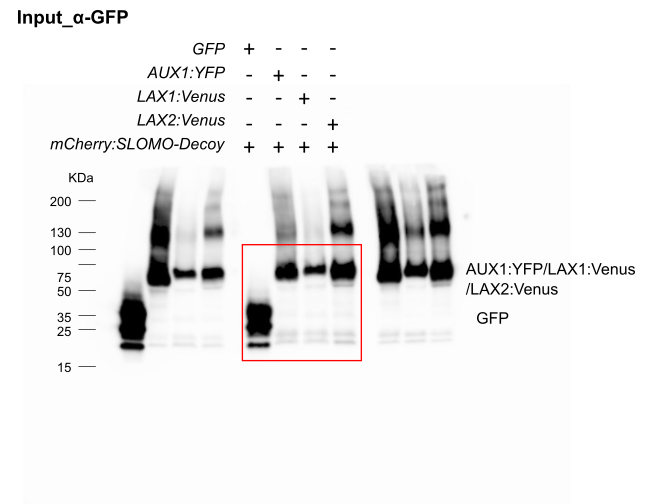

Supplement: Supplementary file 4 — Source data Fig. 2 [file 44318_2026_746_MOESM4_ESM.zip › Figure 2 Source Data/Figure 2A/Input_Anti-GFP.tiff]

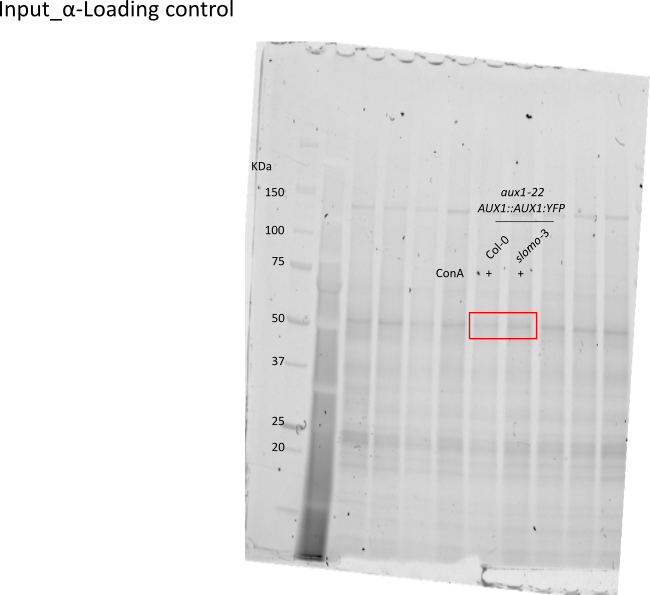

Supplement: Supplementary file 4 — Source data Fig. 2 [file 44318_2026_746_MOESM4_ESM.zip › Figure 2 Source Data/Figure 2C/Rep3/Input_Loading control.tiff]

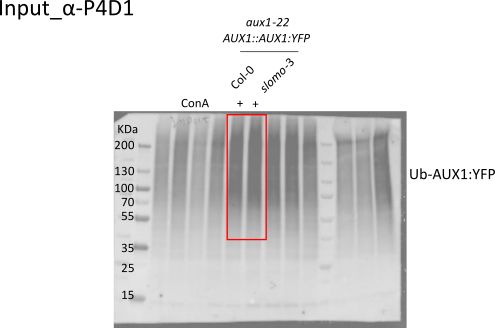

Supplement: Supplementary file 4 — Source data Fig. 2 [file 44318_2026_746_MOESM4_ESM.zip › Figure 2 Source Data/Figure 2C/Rep3/Input_Anti_P4D1.tiff]

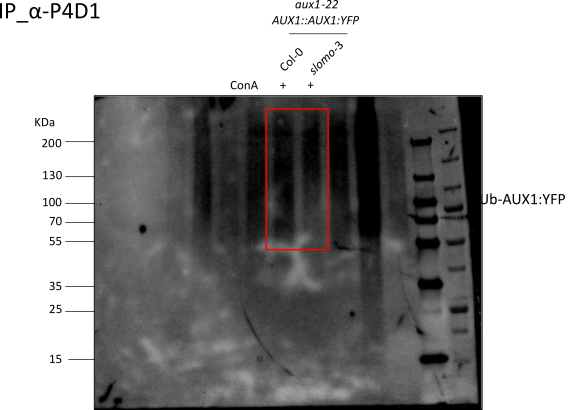

Supplement: Supplementary file 4 — Source data Fig. 2 [file 44318_2026_746_MOESM4_ESM.zip › Figure 2 Source Data/Figure 2C/Rep3/IP_anti_P4D1.tiff]

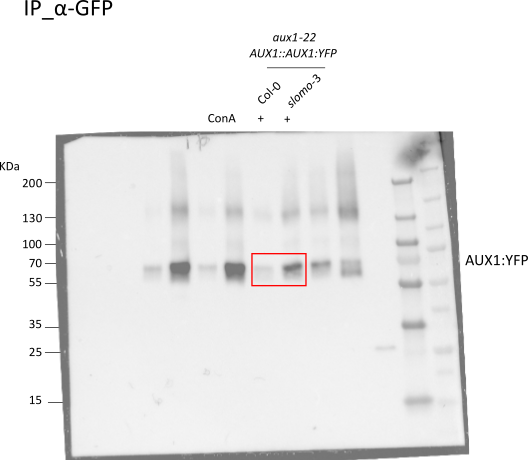

Supplement: Supplementary file 4 — Source data Fig. 2 [file 44318_2026_746_MOESM4_ESM.zip › Figure 2 Source Data/Figure 2C/Rep3/IP_Anti_GFP.tiff]

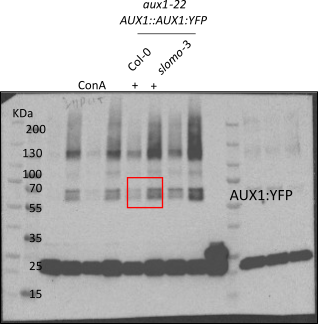

Supplement: Supplementary file 4 — Source data Fig. 2 [file 44318_2026_746_MOESM4_ESM.zip › Figure 2 Source Data/Figure 2C/Rep3/Input_Anti_GFP.tiff]

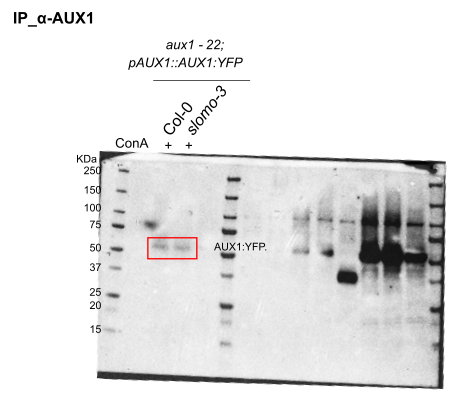

Supplement: Supplementary file 4 — Source data Fig. 2 [file 44318_2026_746_MOESM4_ESM.zip › Figure 2 Source Data/Figure 2C/Rep1/IP_Anti-AUX1.tiff]

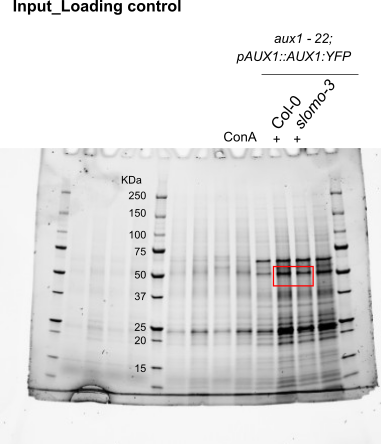

Supplement: Supplementary file 4 — Source data Fig. 2 [file 44318_2026_746_MOESM4_ESM.zip › Figure 2 Source Data/Figure 2C/Rep1/Input_Loading control.tiff]

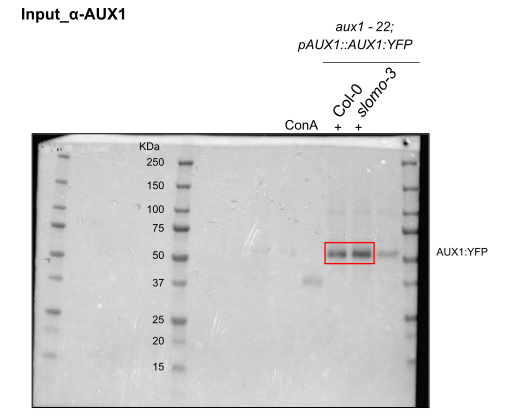

Supplement: Supplementary file 4 — Source data Fig. 2 [file 44318_2026_746_MOESM4_ESM.zip › Figure 2 Source Data/Figure 2C/Rep1/Input_Anti-AUX1.tiff]

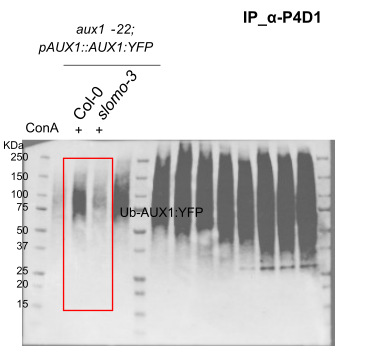

Supplement: Supplementary file 4 — Source data Fig. 2 [file 44318_2026_746_MOESM4_ESM.zip › Figure 2 Source Data/Figure 2C/Rep1/IP_Anti-P4D1.tiff]

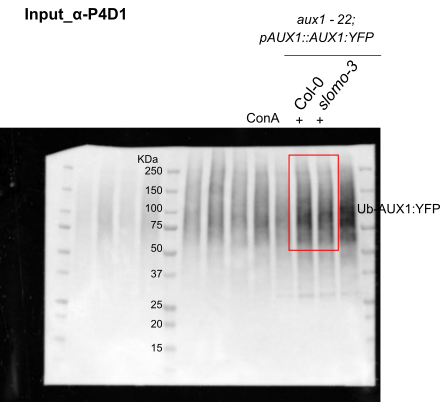

Supplement: Supplementary file 4 — Source data Fig. 2 [file 44318_2026_746_MOESM4_ESM.zip › Figure 2 Source Data/Figure 2C/Rep1/Input_Anti-P4D1.tiff]

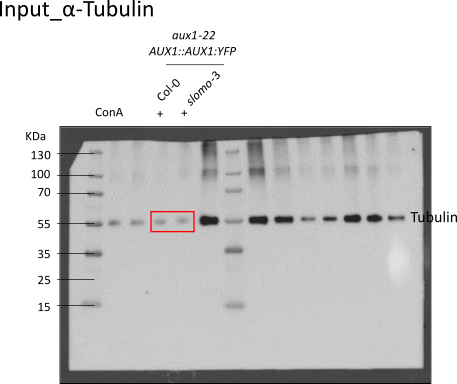

Supplement: Supplementary file 4 — Source data Fig. 2 [file 44318_2026_746_MOESM4_ESM.zip › Figure 2 Source Data/Figure 2C/Rep2/Input_Anti_Tubulin.tiff]

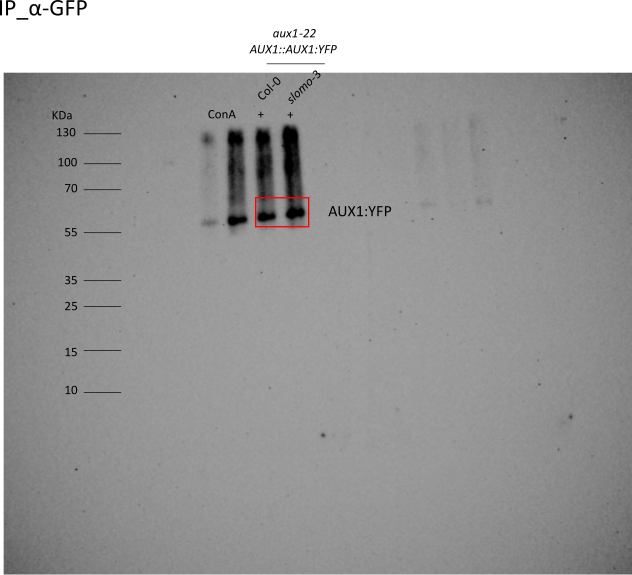

Supplement: Supplementary file 4 — Source data Fig. 2 [file 44318_2026_746_MOESM4_ESM.zip › Figure 2 Source Data/Figure 2C/Rep2/IP_Anti_GFP.tiff]

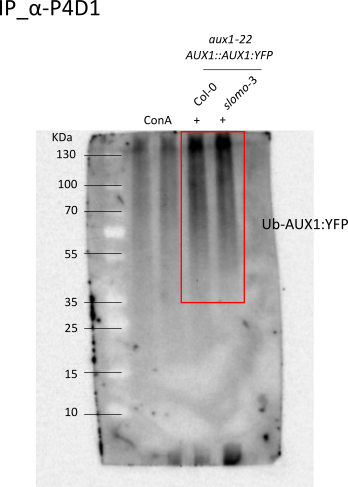

Supplement: Supplementary file 4 — Source data Fig. 2 [file 44318_2026_746_MOESM4_ESM.zip › Figure 2 Source Data/Figure 2C/Rep2/IP_P4D1.tiff]

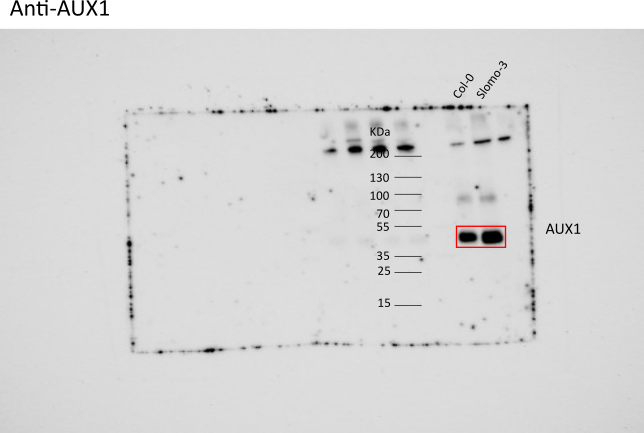

Supplement: Supplementary file 5 — Source data Fig. 3 [file 44318_2026_746_MOESM5_ESM.zip › Figure 3 Source Data/Figure 3A/Rep3/Anti_AUX1.tiff]

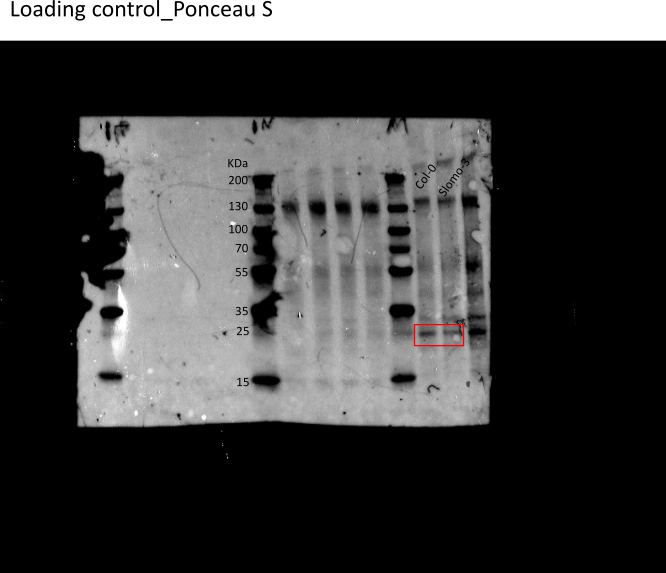

Supplement: Supplementary file 5 — Source data Fig. 3 [file 44318_2026_746_MOESM5_ESM.zip › Figure 3 Source Data/Figure 3A/Rep3/Loading control.tiff]

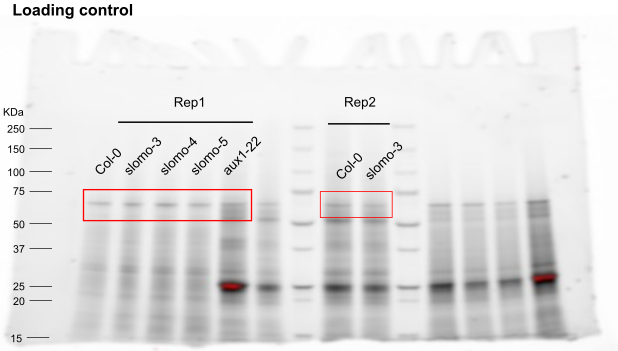

Supplement: Supplementary file 5 — Source data Fig. 3 [file 44318_2026_746_MOESM5_ESM.zip › Figure 3 Source Data/Figure 3A/Rep1-2/Loading control.tiff]

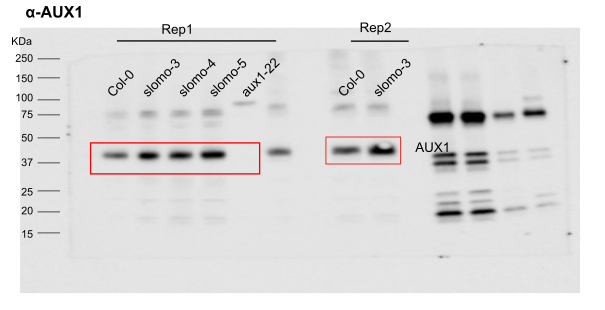

Supplement: Supplementary file 5 — Source data Fig. 3 [file 44318_2026_746_MOESM5_ESM.zip › Figure 3 Source Data/Figure 3A/Rep1-2/Anti-AUX1.tiff]

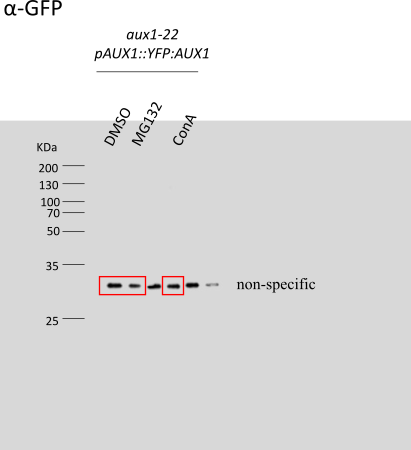

Supplement: Supplementary file 5 — Source data Fig. 3 [file 44318_2026_746_MOESM5_ESM.zip › Figure 3 Source Data/Figure 3G/Rep3/Loading control.tiff]

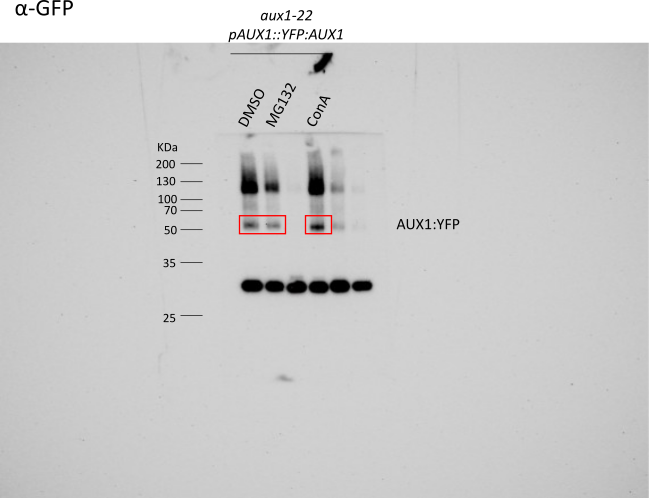

Supplement: Supplementary file 5 — Source data Fig. 3 [file 44318_2026_746_MOESM5_ESM.zip › Figure 3 Source Data/Figure 3G/Rep3/Anti-GFP.tiff]

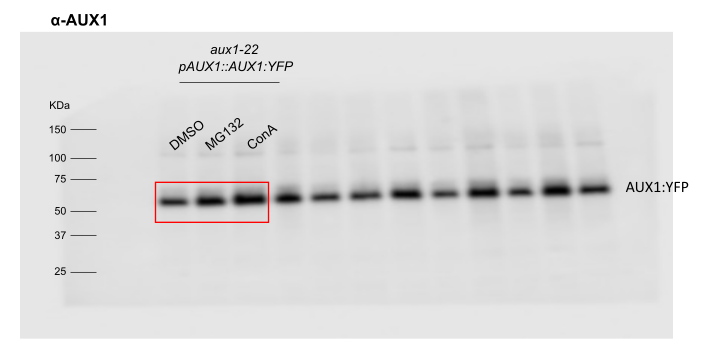

Supplement: Supplementary file 5 — Source data Fig. 3 [file 44318_2026_746_MOESM5_ESM.zip › Figure 3 Source Data/Figure 3G/Rep1/Anti-AUX1.tiff]

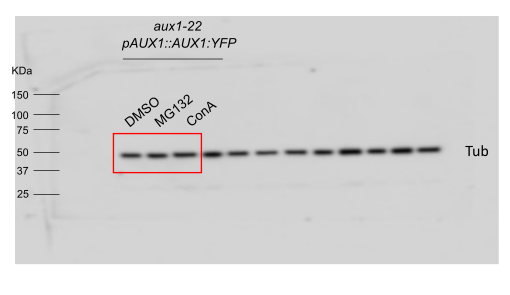

Supplement: Supplementary file 5 — Source data Fig. 3 [file 44318_2026_746_MOESM5_ESM.zip › Figure 3 Source Data/Figure 3G/Rep1/Loading control_α-Tub.tiff]

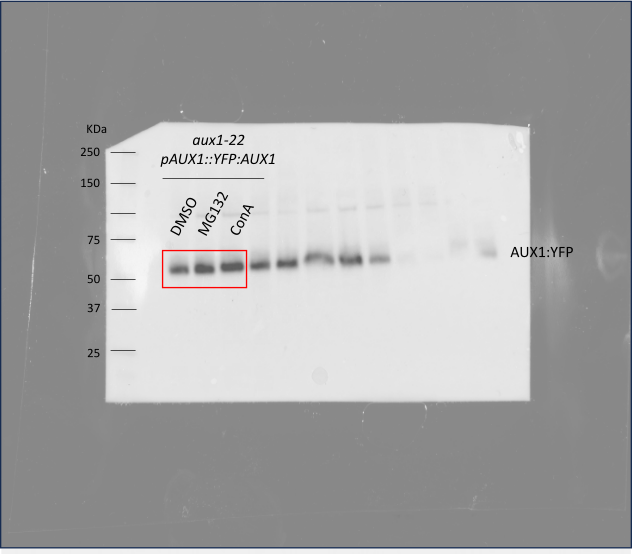

Supplement: Supplementary file 5 — Source data Fig. 3 [file 44318_2026_746_MOESM5_ESM.zip › Figure 3 Source Data/Figure 3G/Rep2/Anti_AUX1.tiff]

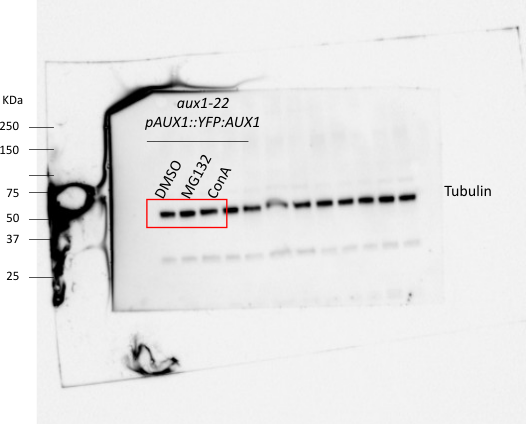

Supplement: Supplementary file 5 — Source data Fig. 3 [file 44318_2026_746_MOESM5_ESM.zip › Figure 3 Source Data/Figure 3G/Rep2/Loading control.tiff]

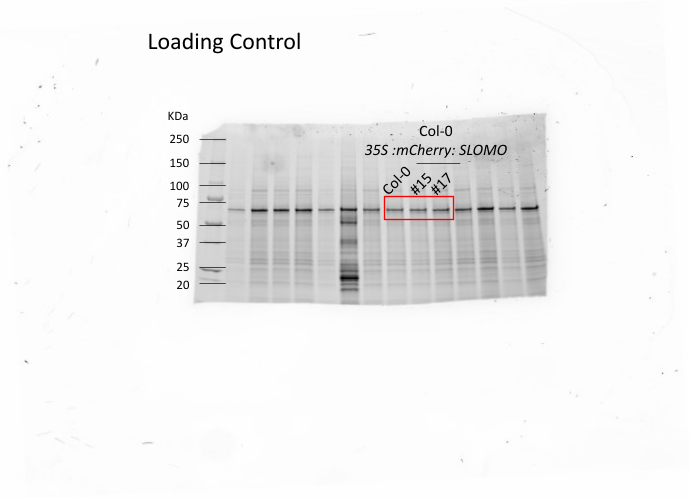

Supplement: Supplementary file 5 — Source data Fig. 3 [file 44318_2026_746_MOESM5_ESM.zip › Figure 3 Source Data/Figure 3E/Rep3/Loading control.tiff]

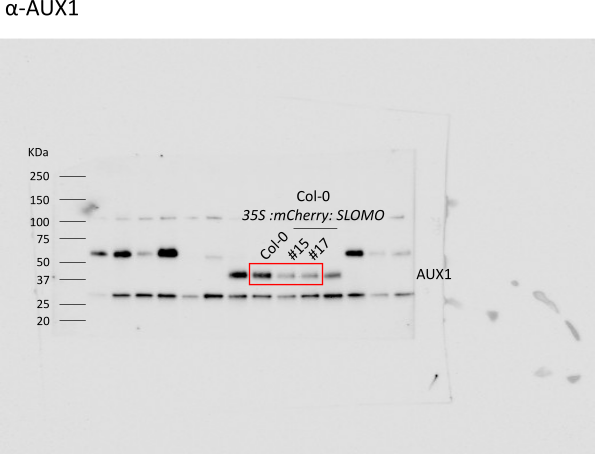

Supplement: Supplementary file 5 — Source data Fig. 3 [file 44318_2026_746_MOESM5_ESM.zip › Figure 3 Source Data/Figure 3E/Rep3/Anti-AUX1.tiff]

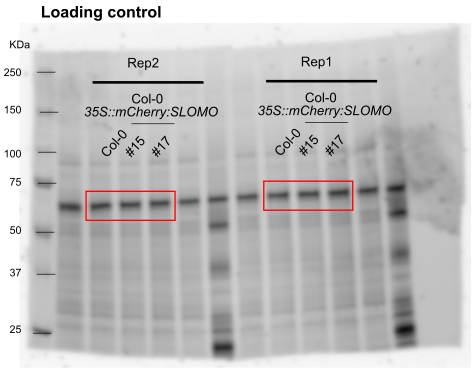

Supplement: Supplementary file 5 — Source data Fig. 3 [file 44318_2026_746_MOESM5_ESM.zip › Figure 3 Source Data/Figure 3E/Rep1-2/Loading control.tiff]

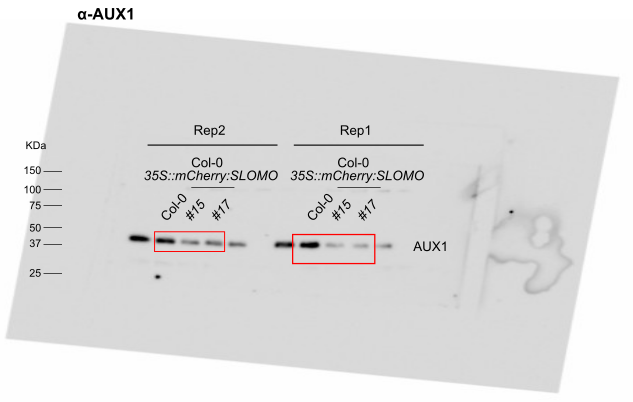

Supplement: Supplementary file 5 — Source data Fig. 3 [file 44318_2026_746_MOESM5_ESM.zip › Figure 3 Source Data/Figure 3E/Rep1-2/Anti-AUX1 .tiff]

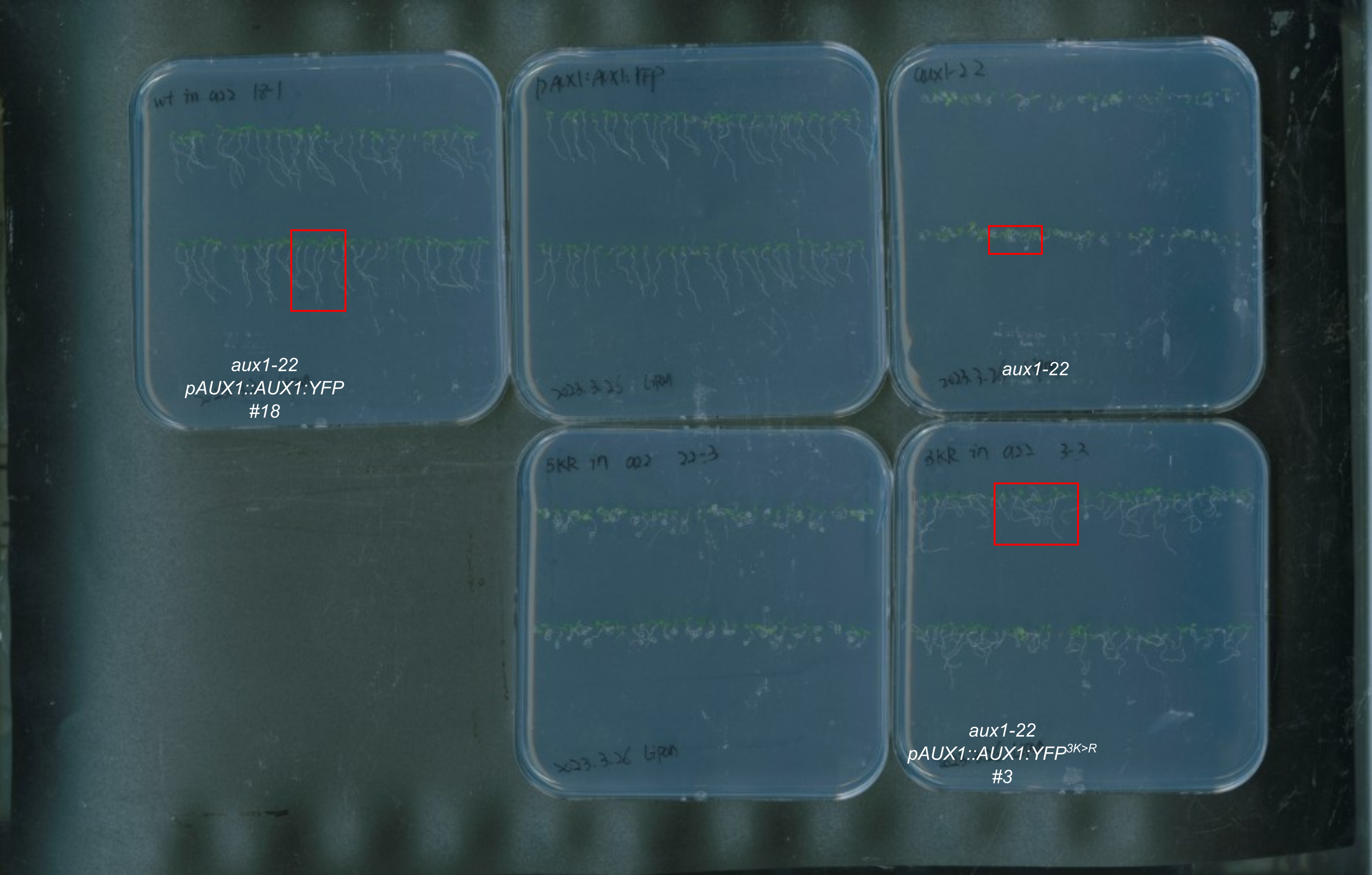

Supplement: Supplementary file 6 — Source data Fig. 4 [file 44318_2026_746_MOESM6_ESM.zip › Figure 4 Source Data/Figure 4H/Figure 4H.tiff]

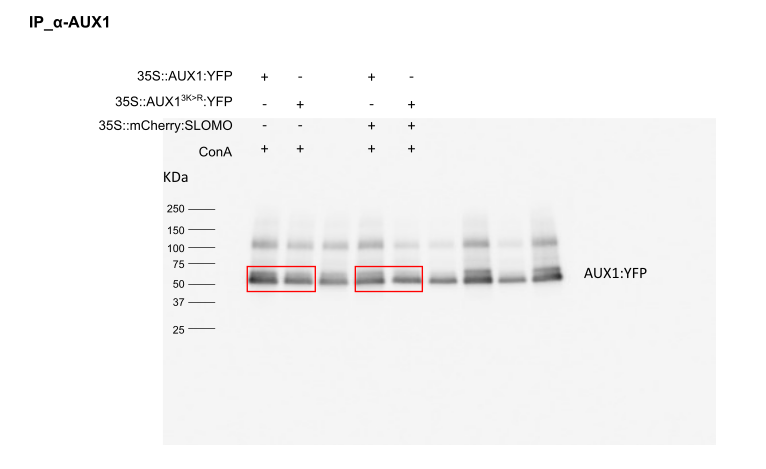

Supplement: Supplementary file 6 — Source data Fig. 4 [file 44318_2026_746_MOESM6_ESM.zip › Figure 4 Source Data/Figure 4D/IP_anti-AUX1.tiff]

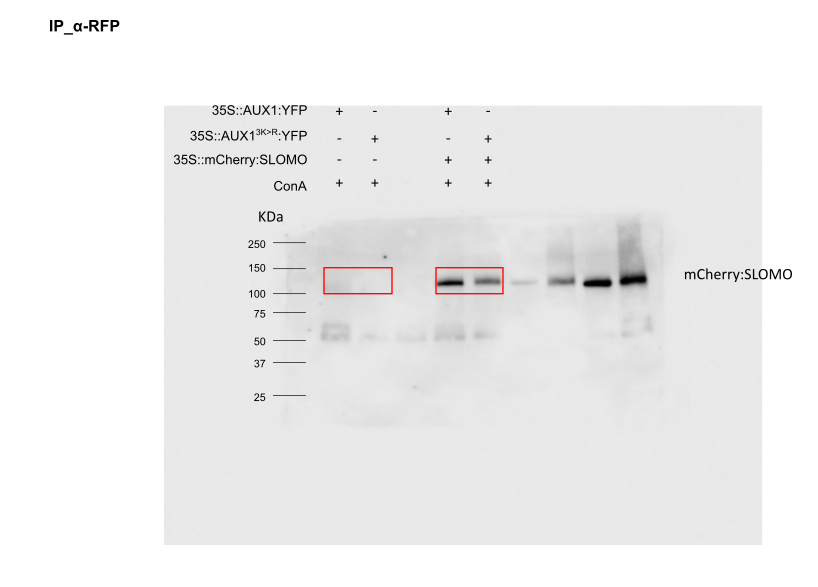

Supplement: Supplementary file 6 — Source data Fig. 4 [file 44318_2026_746_MOESM6_ESM.zip › Figure 4 Source Data/Figure 4D/IP_anti-RFP.tiff]

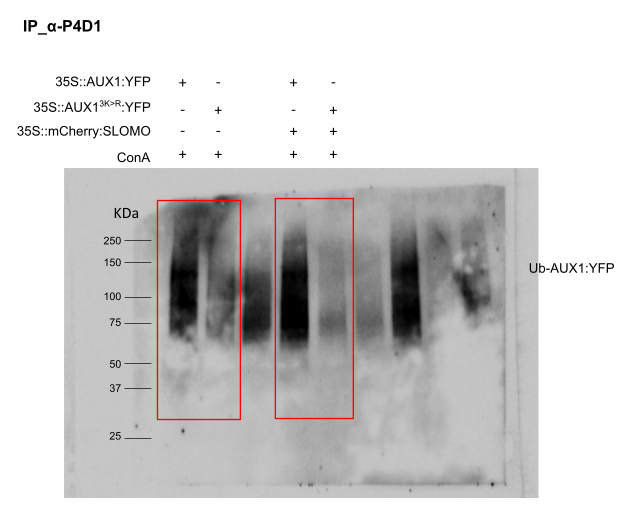

Supplement: Supplementary file 6 — Source data Fig. 4 [file 44318_2026_746_MOESM6_ESM.zip › Figure 4 Source Data/Figure 4D/IP_anti-P4D1.tiff]

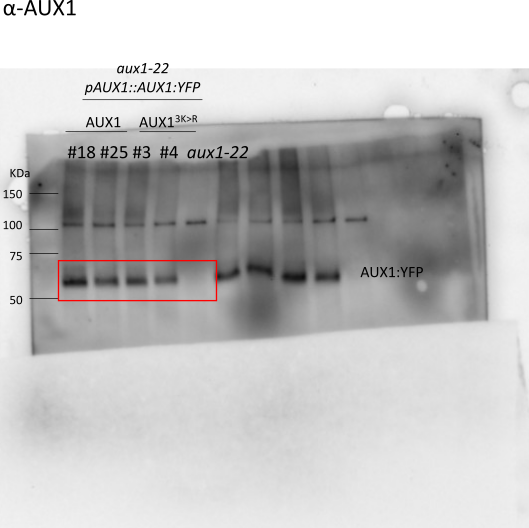

Supplement: Supplementary file 6 — Source data Fig. 4 [file 44318_2026_746_MOESM6_ESM.zip › Figure 4 Source Data/Figure 4F/Rep3/Anti_AUX1.tiff]

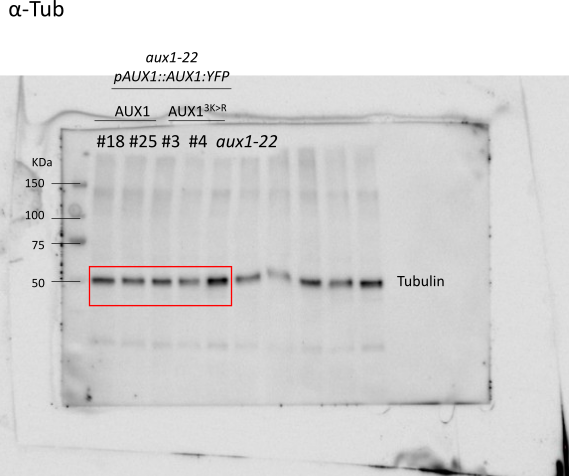

Supplement: Supplementary file 6 — Source data Fig. 4 [file 44318_2026_746_MOESM6_ESM.zip › Figure 4 Source Data/Figure 4F/Rep3/Loading control.tiff]

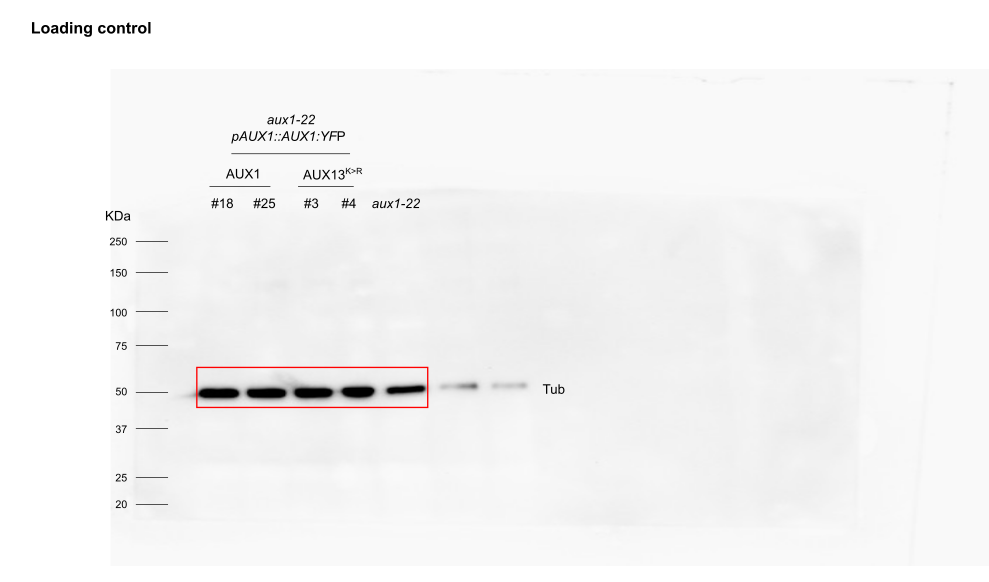

Supplement: Supplementary file 6 — Source data Fig. 4 [file 44318_2026_746_MOESM6_ESM.zip › Figure 4 Source Data/Figure 4F/Rep1/Loading control.tiff]

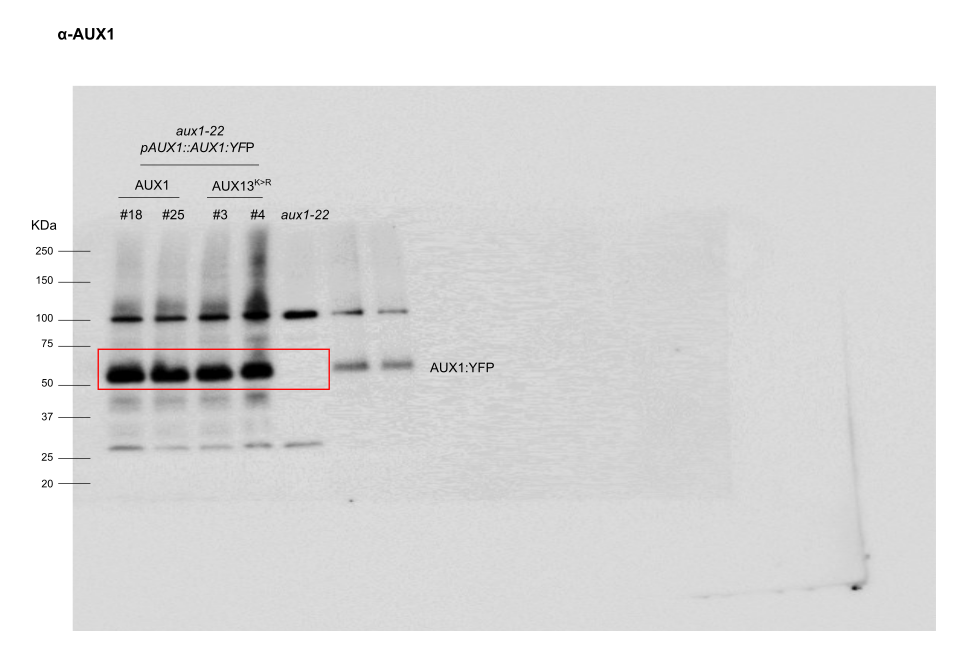

Supplement: Supplementary file 6 — Source data Fig. 4 [file 44318_2026_746_MOESM6_ESM.zip › Figure 4 Source Data/Figure 4F/Rep1/anti-AUX1.tiff]

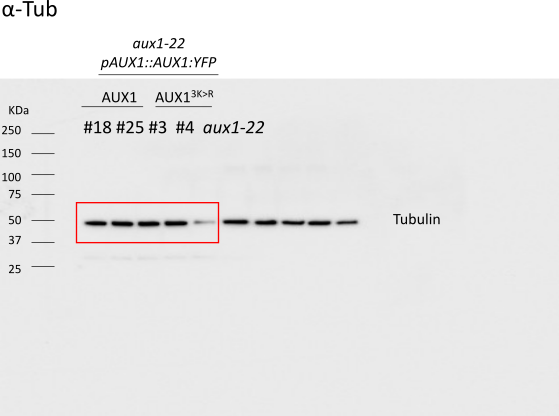

Supplement: Supplementary file 6 — Source data Fig. 4 [file 44318_2026_746_MOESM6_ESM.zip › Figure 4 Source Data/Figure 4F/Rep2/Loading control.tiff]

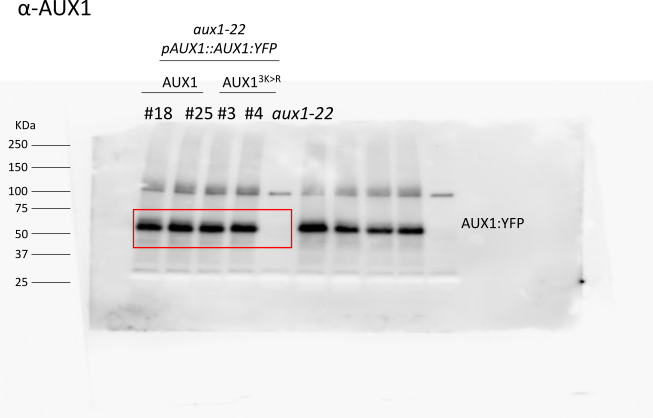

Supplement: Supplementary file 6 — Source data Fig. 4 [file 44318_2026_746_MOESM6_ESM.zip › Figure 4 Source Data/Figure 4F/Rep2/Anti-AUX1.tiff]

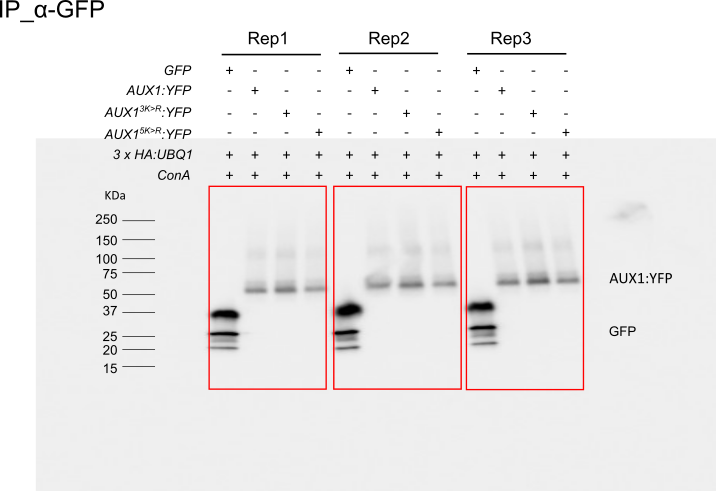

Supplement: Supplementary file 6 — Source data Fig. 4 [file 44318_2026_746_MOESM6_ESM.zip › Figure 4 Source Data/Figure 4B/IP_anti-GFP.tiff]

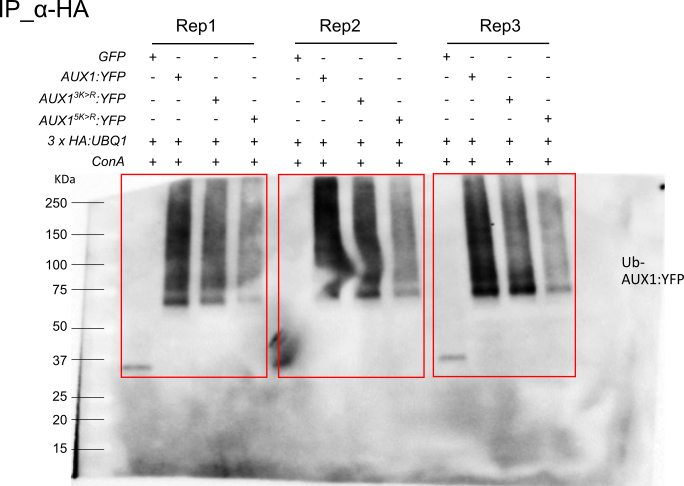

Supplement: Supplementary file 6 — Source data Fig. 4 [file 44318_2026_746_MOESM6_ESM.zip › Figure 4 Source Data/Figure 4B/IP_anti-HA.tiff]

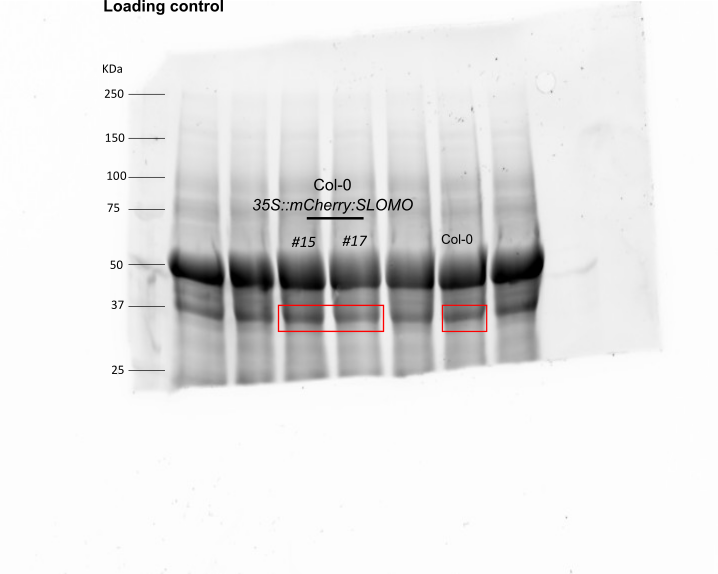

Supplement: Supplementary file 8 — Appendix Figures Source Data [file 44318_2026_746_MOESM8_ESM.zip › Appendix Figure S3 Source Data/Appendix Figure S3b/loading control.tiff]

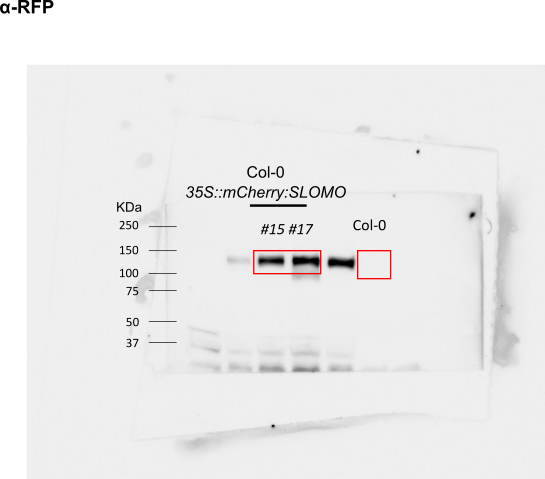

Supplement: Supplementary file 8 — Appendix Figures Source Data [file 44318_2026_746_MOESM8_ESM.zip › Appendix Figure S3 Source Data/Appendix Figure S3b/Anti-RFP.tiff]

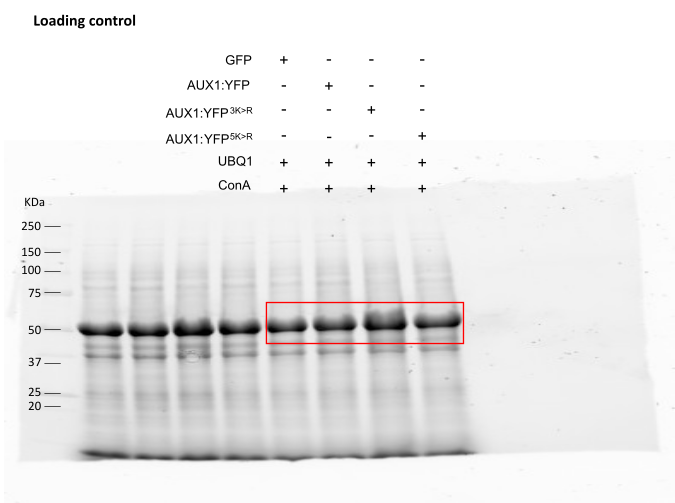

Supplement: Supplementary file 8 — Appendix Figures Source Data [file 44318_2026_746_MOESM8_ESM.zip › Appendix Figure S18 Source Data/Appendix Figure S18b/Input_Loading control.tiff]

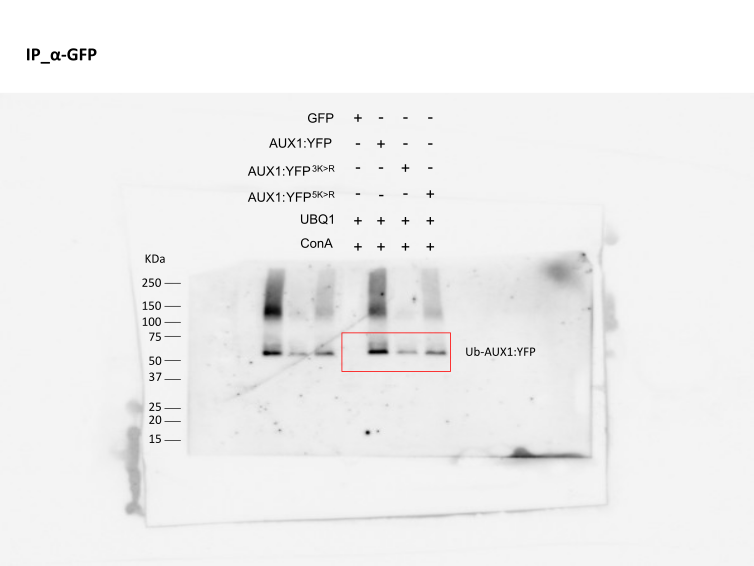

Supplement: Supplementary file 8 — Appendix Figures Source Data [file 44318_2026_746_MOESM8_ESM.zip › Appendix Figure S18 Source Data/Appendix Figure S18b/IP_anti-GFP.tiff]

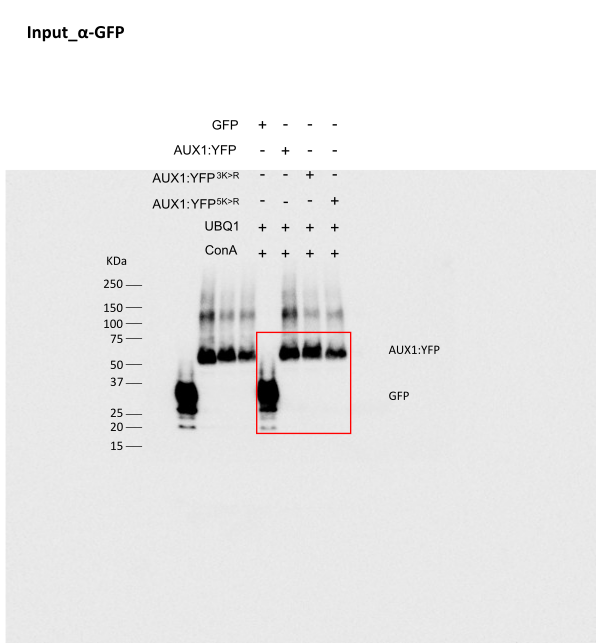

Supplement: Supplementary file 8 — Appendix Figures Source Data [file 44318_2026_746_MOESM8_ESM.zip › Appendix Figure S18 Source Data/Appendix Figure S18b/Input_Anti-GFP.tiff]

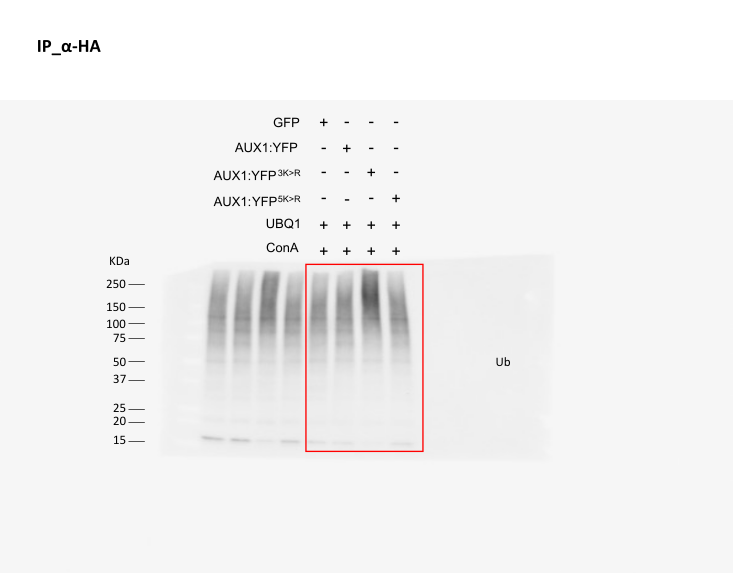

Supplement: Supplementary file 8 — Appendix Figures Source Data [file 44318_2026_746_MOESM8_ESM.zip › Appendix Figure S18 Source Data/Appendix Figure S18b/IP_Anti-HA.tiff]

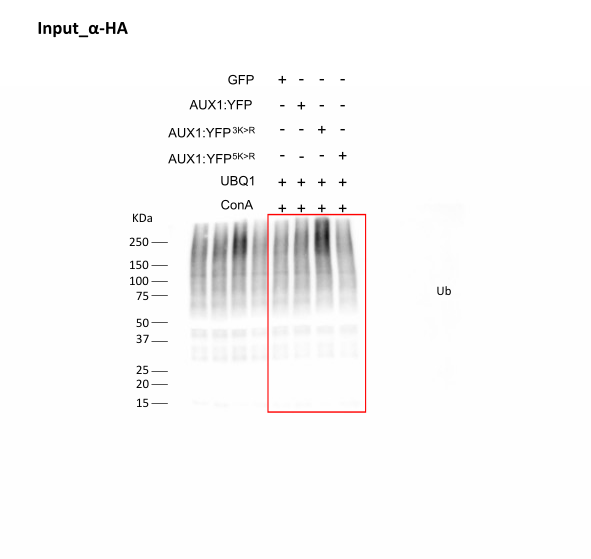

Supplement: Supplementary file 8 — Appendix Figures Source Data [file 44318_2026_746_MOESM8_ESM.zip › Appendix Figure S18 Source Data/Appendix Figure S18b/Input_Anti-HA.tiff]

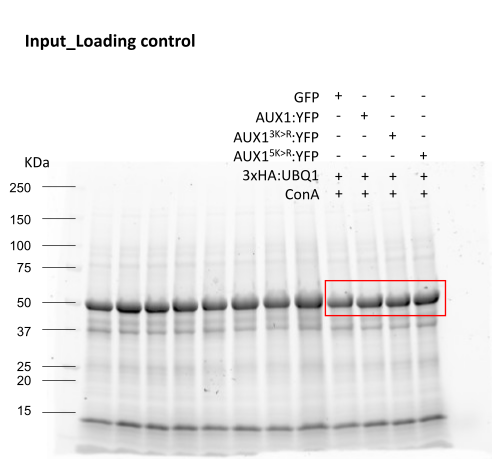

Supplement: Supplementary file 8 — Appendix Figures Source Data [file 44318_2026_746_MOESM8_ESM.zip › Appendix Figure S18 Source Data/Appendix Figure S18a/Loading control.tiff]

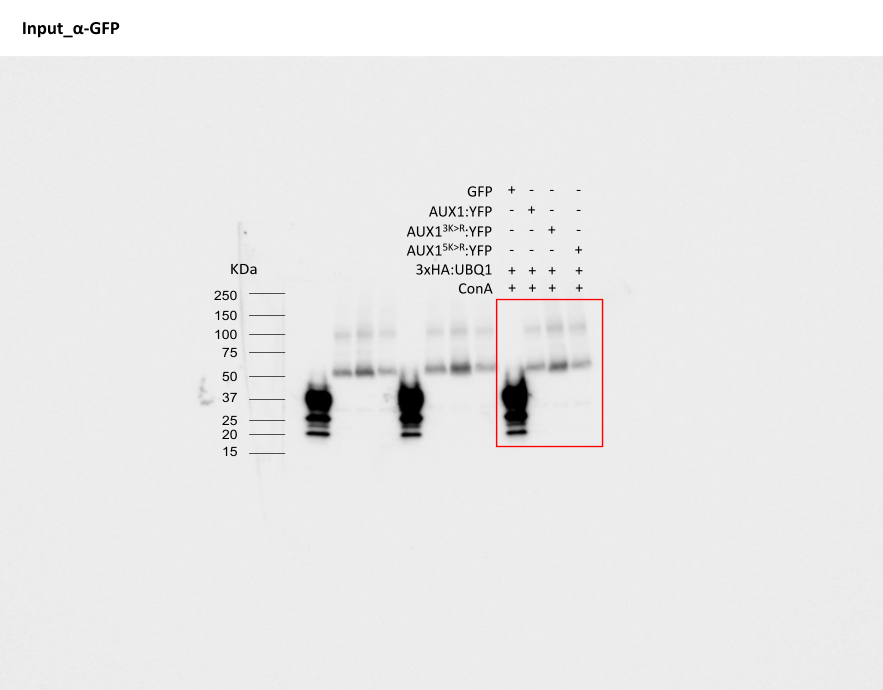

Supplement: Supplementary file 8 — Appendix Figures Source Data [file 44318_2026_746_MOESM8_ESM.zip › Appendix Figure S18 Source Data/Appendix Figure S18a/Input_anti-GFP.tiff]

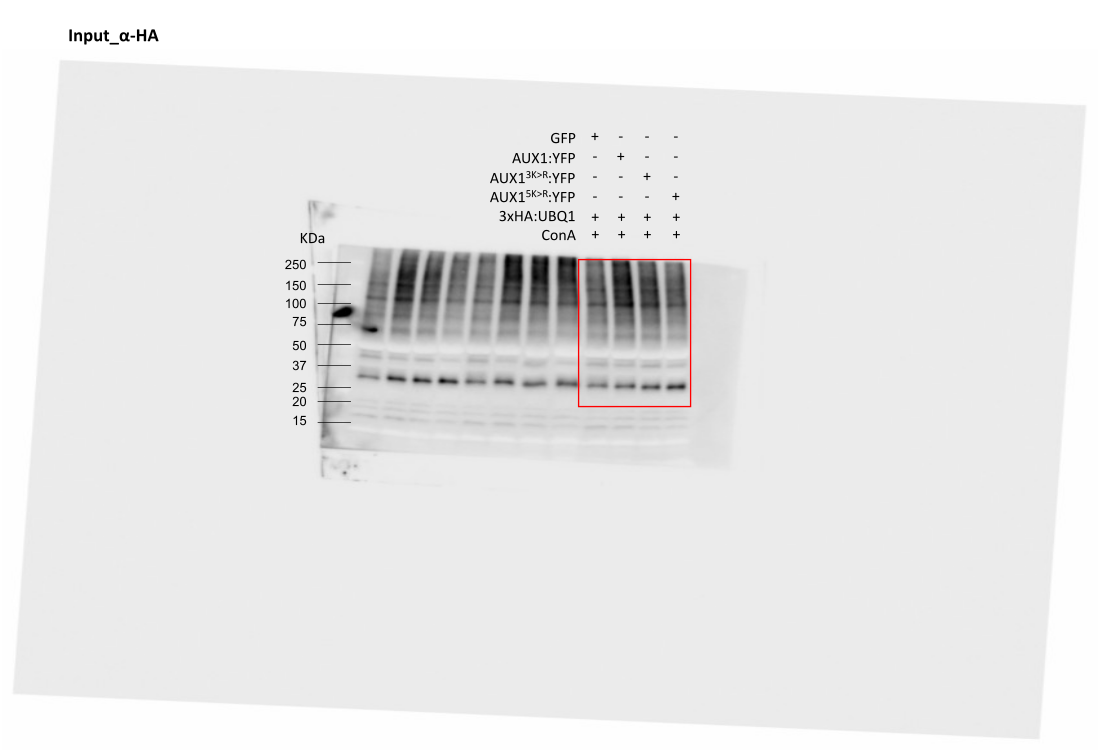

Supplement: Supplementary file 8 — Appendix Figures Source Data [file 44318_2026_746_MOESM8_ESM.zip › Appendix Figure S18 Source Data/Appendix Figure S18a/Input anti-HA.tiff]

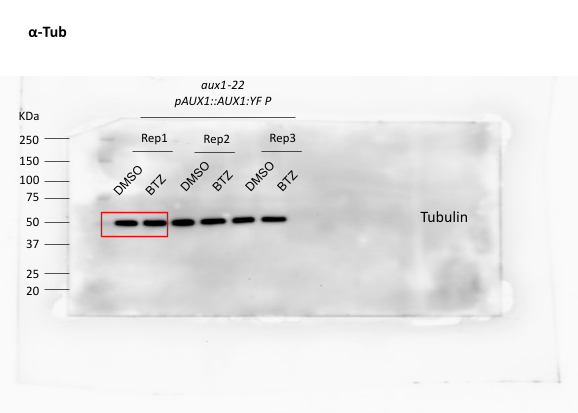

Supplement: Supplementary file 8 — Appendix Figures Source Data [file 44318_2026_746_MOESM8_ESM.zip › Appendix Figure S14 Source Data/Appendix Figure S14a/Loading control.tiff]

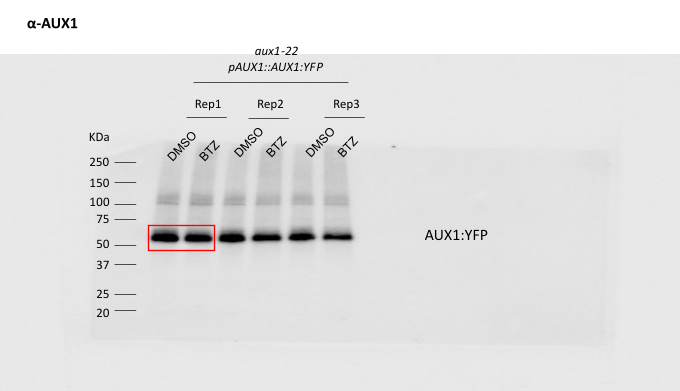

Supplement: Supplementary file 8 — Appendix Figures Source Data [file 44318_2026_746_MOESM8_ESM.zip › Appendix Figure S14 Source Data/Appendix Figure S14a/Anti-AUX1.tiff]

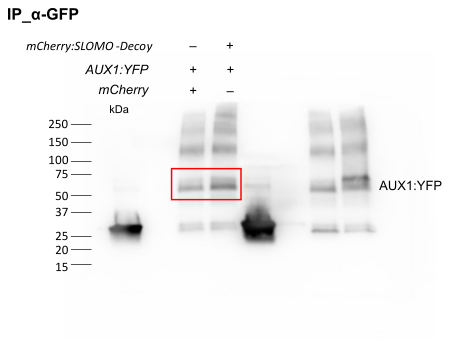

Supplement: Supplementary file 8 — Appendix Figures Source Data [file 44318_2026_746_MOESM8_ESM.zip › Appendix Figure S9 Source Data/IP_Anti-GFP.tiff]

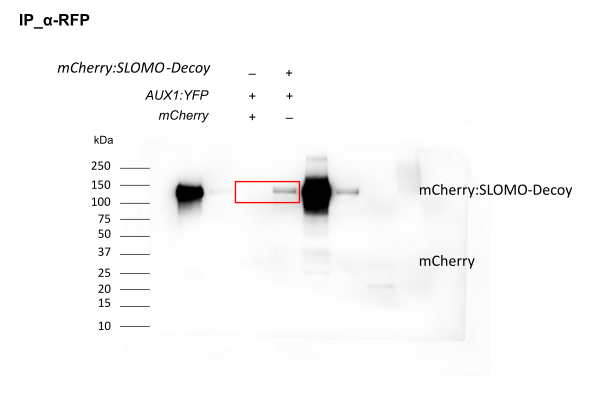

Supplement: Supplementary file 8 — Appendix Figures Source Data [file 44318_2026_746_MOESM8_ESM.zip › Appendix Figure S9 Source Data/IP_Anti-RFP.tiff]

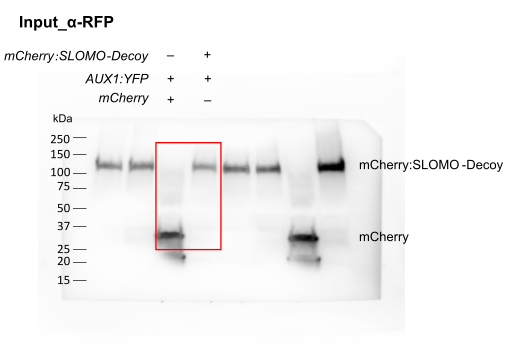

Supplement: Supplementary file 8 — Appendix Figures Source Data [file 44318_2026_746_MOESM8_ESM.zip › Appendix Figure S9 Source Data/Input_Anti-RFP.tiff]

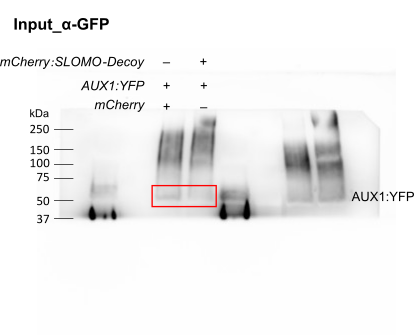

Supplement: Supplementary file 8 — Appendix Figures Source Data [file 44318_2026_746_MOESM8_ESM.zip › Appendix Figure S9 Source Data/Input_Anti-GFP.tiff]
